# *DOR/Tp53inp2* and *Tp53inp1* Constitute a Metazoan Gene Family Encoding Dual Regulators of Autophagy and Transcription

**DOI:** 10.1371/journal.pone.0034034

**Published:** 2012-03-28

**Authors:** Ana Sancho, Jordi Duran, Antonio García-España, Caroline Mauvezin, Endalkachew A. Alemu, Trond Lamark, Maria J. Macias, Rob DeSalle, Miriam Royo, David Sala, Javier U. Chicote, Manuel Palacín, Terje Johansen, Antonio Zorzano

**Affiliations:** 1 Institute for Research in Biomedicine (IRB Barcelona), Barcelona, Spain; 2 Departament de Bioquímica i Biologia Molecular, Facultat de Biologia, Universitat de Barcelona, Barcelona, Spain; 3 CIBER de Diabetes y Enfermedades Metabólicas Asociadas (CIBERDEM), Instituto de Salud Carlos III, Madrid, Spain; 4 Unitat de Recerca, Hospital Joan XXIII, Institut de Investigacio Sanitaria Rovira I Virgili (IISPV), Universitat Rovira i Virgili, Tarragona, Spain; 5 Molecular Cancer Research Group, Institute of Medical Biology, University of Tromsø, Tromsø, Norway; 6 ICREA, Institució Catalana de Recerca i Estudis Avançats, Barcelona, Spain; 7 Sackler Institute for Comparative Genomics, American Museum of Natural History, New York, New York, United States of America; 8 Combinatorial Chemistry Unit, Barcelona Science Park, Barcelona, Spain; Boston University, United States of America

## Abstract

Human DOR/TP53INP2 displays a unique bifunctional role as a modulator of autophagy and gene transcription. However, the domains or regions of DOR that participate in those functions have not been identified. Here we have performed structure/function analyses of DOR guided by identification of conserved regions in the DOR gene family by phylogenetic reconstructions. We show that DOR is present in metazoan species. Invertebrates harbor only one gene, *DOR/Tp53inp2*, and in the common ancestor of vertebrates *Tp53inp1* may have arisen by gene duplication. In keeping with these data, we show that human TP53INP1 regulates autophagy and that different DOR/TP53INP2 and TP53INP1 proteins display transcriptional activity. The use of molecular evolutionary information has been instrumental to determine the regions that participate in DOR functions. DOR and TP53INP1 proteins share two highly conserved regions (region 1, aa residues 28–42; region 2, 66–112 in human DOR). Mutation of conserved hydrophobic residues in region 1 of DOR (that are part of a nuclear export signal, NES) reduces transcriptional activity, and blocks nuclear exit and autophagic activity under autophagy-activated conditions. We also identify a functional and conserved LC3-interacting motif (LIR) in region 1 of DOR and TP53INP1 proteins. Mutation of conserved acidic residues in region 2 of DOR reduces transcriptional activity, impairs nuclear exit in response to autophagy activation, and disrupts autophagy. Taken together, our data reveal DOR and TP53INP1 as dual regulators of transcription and autophagy, and identify two conserved regions in the DOR family that concentrate multiple functions crucial for autophagy and transcription.

## Introduction

Macroautophagy (here referred to as autophagy) is a major cellular pathway for the degradation of long-lived proteins and organelles [1], [2]. Autophagy is a conserved catabolic cellular process in which macromolecules and organelles are degraded, either as a means to eliminate long-lived, damaged, or faulty cellular components, or in response to stress, such as hypoxia, endoplasmic reticulum stress, or nutrient deprivation. Although our understanding of autophagy has increased substantially in recent years, there are still many open questions related to the identity of the components of the autophagic pathway, the molecular signals that regulate it, and the mechanisms that target specific cellular components to be degraded. In this respect, there is evidence of a bidirectional functional link between nuclear regulators of gene transcription and autophagy. Factors such as p53 or E2F1 are activated by DNA damage and stimulate autophagy through transcriptional events [3], [4], [5], [6], [7], [8]. Moreover, some autophagic proteins, such as Beclin 1, p62 or LC3, undergo nucleocytoplasmic shuttling in cells [9], [10], [11], which may be relevant to the regulation of autophagy.

The nuclear cofactor DOR (Diabetes- and Obesity-Regulated gene, also known as Tp53inp2 or C20orf110), is a key regulator of autophagy [12], [13]. Originally identified as a protein highly expressed in muscle, heart and brain, DOR localizes in nuclear bodies in proliferating cells [14]. In agreement with its nuclear localization, DOR functions as an enhancer of the transcriptional activity of the thyroid hormone receptor TRα1, and DOR loss-of-function reduces thyroid hormone function in muscle cells [14]. This function is driven mainly by the N-terminal part of the protein, whereas the C-terminal fragment of DOR exhibits inhibitory activity [14]. Moreover, DOR physically interacts with TRα1 and T_3_-responsive promoters, as assessed by co-immunoprecipitation and ChIP assays [14]. The Drosophila homolog of DOR, dDOR, is a coactivator of the ecdysone receptor [15]. dDOR binds the ecdysone receptor and is required for its maximal transcriptional activity. In the absence of dDOR, flies display a number of ecdysone loss-of-function phenotypes such as impaired spiracle eversion, impaired salivary gland degradation, and pupal lethality [15].

In response to cellular stress or activation of autophagy, DOR exits the nucleus [12], [13] and relocates to early autophagosomes [12]. Thus, DOR interacts directly with the autophagosome-membrane-associated proteins LC3 and GATE16 and regulates autophagy in mammalian cells and in Drosophila [12]. HeLa cells transiently transfected with DOR show an increase in protein degradation rates and have higher numbers of GFP-LC3-positive puncta per cell, as well as an increased accumulation of autophagosomes, both under basal and starvation conditions [12]. In contrast, DOR loss-of-function in muscle cells leads to fewer GFP-LC3-positive puncta, fewer autophagosomes, and substantial inhibition of protein degradation compared with control cells [12]. Silencing of dDOR in larval fat bodies caused pupae to undergo late-stage cell death, larvae in the wandering stage had low number of Lysotracker-positive puncta in the fat body, as well as less autolysosomes compared with control larvae [12]. On the basis of these studies, we analyzed the conservation of the DOR/TP53INP2 sequences using phylogenetic reconstructions. Our findings indicated conservation among metazoans but not in other kingdoms, and revealed proteins in metazoans with potential regulatory properties similar to those of DOR, such as TP53INP1. In addition, subsequent analysis of sequences also revealed two highly conserved regions that contain signals which we demonstrate here to be essential for the functional properties of DOR and TP53INP1 in autophagy and gene transcription.

## Methods

### Data mining and sequence analysis

All the sequences used in this study are listed in Table S1. Data mining was performed as previously described [16], [17]. Blast searches with the Blast-T program were performed with known DOR/TP53INP1 sequences as multiple starting queries. We searched various genome-sequencing projects at the NCBI (www.ncbi.nlm.nih.gov), the Ensembl (www.ensembl.org) or the JGI (http://www.jgi.doe.gov/) servers; and the translated nucleotide and protein databases at NCBI. Intron-exon borders were determined or verified as previously reported [18]. Alignments of protein sequences were performed using the ClustalW or the Mulialin programmes at the NPS@: Network Protein Sequence Analysis (http://npsa-pbil.ibcp.fr/cgi-bin/npsa_automat.pl?page=/NPSA/npsa_server.html). Identities between protein sequences were calculated with the Dialign software from the GenomatixSuite (www.genomatix.de/cgi-bin/dialign/dialign.pl). For phylogenetic analyses, the protein sequences were aligned using MAFFT and the BLOSSUM 62 matrix with other settings on default. Phylogenetic trees were generated using parsimony [17], and Maximum Likelihood [19]. Parsimony was performed using all characters as unordered and the Gonnett cost matrix. For each analysis, jackknife proportions were computed in PAUP* (Swofford 2003) using 100 jackknife replicates. Likelihood analyses were performed using the WAG assumptions in RaxML. Bootstrap values for likelihood were computed in RaxML using 100 replicates.

### Plasmids

The following vectors were used: pENTR1A (Invitrogen; Entry vector) and pDest53 (Invitrogen; Mammalian GFP fusion expression vector). The following cDNA constructs have been described previously: pDest15-LC3A, pDest15-LC3B, pDest15-GABARAP, pDest15-GABARAP-L1, and pDest15-GABARAP-L2 [20]; pGEX-4T-1-hMAP1LC3C (ΔG), pDest15-LC3B (1–28), pDest15-LC3B (30–125), pDest15-LC3B (R10A/R11A), pDest15-LC3B (R70A), pDest15-LC3B (F52A), pDest15-LC3B (L53A), pDest15-LC3B (G120A), and pDest15-LC3B(1–120) [21]. hDOR (BC035639) and hTP53INP1 (BC074868) were amplified by PCR from IMAGE clones and subcloned into Gateway entry vectors by traditional cloning. DOR (aa. 27–46) and TP53INP1 (aa. 23–42) were PCR-amplified from pENTR-DOR and pENTR-TP53INP1, respectively, and then subcloned into Gateway entry vectors. pENTR-DOR (W35A/I38A) and pENTR-TP53INP1(W31A/V34A) were made by introducing the point mutations into pENTR-DOR and pENTR-TP53INP1, respectively, using the QuickChange site-directed mutagenesis kit (Stratagene). Gateway destination plasmids pDest53-DOR, pDest53-DOR (aa 27–46), pDest53-DOR (W35A/I38A), pDest53-TP53INP1, pDest53-TP53INP1 (aa 23–42), and pDest53-TP53INP1(W31A/V34A) were made using Gateway LR recombination reactions (Invitrogen), following the manufacturer's instructions.

A PCR fragment spanning the murine DOR ORF was cloned into the EcoRV and SalI sites of the pcDNA3 (Invitrogen) vector. The same steps were followed for the DOR from other species. Two mutated versions of DOR (L36A L40A and E97K D98K) were generated by the Quick Change Site-Directed Mutagenesis Kit (Stratagene). Full-length DOR cDNA and cDNA fragments encompassing amino acid residues 1–120, 1–111 were PCR-amplified and cloned with NdeI and BamHI in the pGBKT7 vector containing the DNA binding domain of Gal4 (Clontech) and subsequently cloned in pCDNA3. Ortholog cDNAs and fragments were generated in the same way. All plasmid constructs made in this study were verified by DNA sequencing (BigDye sequencing kit, Applied Biosystems). The oligonucleotides used for mutagenesis, PCR, and DNA sequencing were purchased either from Invitrogen or Operon.

### Cell cultures, cell transfection and transcriptional activation assays

HeLa (ATCC CCL-2) and HEK293T (ATCC CRL-11268) cells were maintained in DMEM supplemented with 10% FBS, 25 mM Hepes, penicillin (100 U/ml) and streptomycin (100 µg/ml). GFP-LC3 stable cell line was kindly provided by Dr N. Mizushima. For transient transfection assays, cells were typically plated onto 24-well plates 24 h prior to transfection by the Lipofectamine 2000 method (Invitrogen), as reported (7). All transient transfections included 10% of the total DNA of expression vector for GFP (pEGFP, Clontech) to normalize for transfection efficiency. In a typical experiment, 150 ng of reporter plasmid, 75 ng of nuclear receptor expression plasmid and 100 to 400 ng of DOR expression vector were transfected. Ligands were dissolved in absolute ethanol (1 µM dexamethasone) or water (1 µM rosiglitazone or 100 nM T3). Sixteen hours after transfection, cells were harvested and cell extracts were analyzed for chloramphenicol acetyltransferase (CAT) expression by a specific CAT-Elisa® kit (Roche) or luciferase assay system (Promega). Transfection efficiency was analyzed by flow cytometric analysis of GFP expression.

The reporter vector used to assay thyroid hormone receptor activation and the expression vector for the rat thyroid hormone receptor TRα1 were as previously described (7). The reporter vectors for GR, PPARγ and VDR, and the expression vectors for the rat GR, human PPARγ and human VDR were gifts from Drs. C. Caelles, D. Haro and A. Aranda, respectively.

### Lentiviral infection and siRNA generation

For *TP53INP1* silencing, 5 different human shRNA sequences (MISSION shRNA clones, Sigma-Aldrich) included in shRNA lentiviral plasmids (pLKO.1-puro) were used. Clone ID, target sequence: TRCN0000128518, CCTCTTAACAGAAATAGCCTT; shRNA lentiviral non-target control plasmid (MISSION® Non-Target shRNA Control Transduction Particles, Sigma-Aldrich) was used as a control. Lentiviruses encoding scrambled and *TP53INP1* shRNA were produced by transient transfection of HEK293T [22]. Briefly, the lentivirus expression plasmid (pLKO.1-puro-shTP53INP1 or control vector), together with pCMV-dR8,74 (helper packaging construct) and pMD2.G (vector encoding for envelope protein) plasmids (kindly provided by Dr. D. Trono from the Ecole Polytechnique Federale de Lausanne, Switzerland), were transiently co-transfected into HEK293T cells by the PEI method. Lentivirus were harvested at 48 and 72 h post-transfection, centrifuged to get rid of cell debris, and then filtered through 0.45 µm cellulose acetate filters followed by ultracentrifugation. For lentivirus infection, HeLa cells were plated at 10.000 cells/ml in 6 well plates, infected with 1∶10 of total obtained *TP53INP1*-shRNA lentivirus or control lentivirus and incubated at 33°C. Twenty four hours after infection, virus containing media was removed, standard culture media was added and incubation continued at 37°C. Puromycin selection (2 µg/ml) was performed two days after infection and resistant cells were amplified for 1–2 additional passages. At 70–80% confluence cells were harvested and lysed.

### Immunofluorescence assays

HeLa cells were transfected either with wild-type DOR, the two DOR mutants (L36A/L40A; E97K/D98K) or wild-type TP53INP1 expression vectors by the PEI method. Thirty six hours post-transfection, cells were treated with 2 µM rapamycin (Calbiochem) for 3 h. Amino acid starvation was induced by incubating the cells for 1 h in Hank's Balanced Salt Solution (HBSS, Gibco) with 10% dialyzed FBS, 2% Hepes and 1% penicillin/streptomycin.

After specific treatments, cells were fixed in 4% paraformaldehyde or methanol and washed twice with PBS. Immunocytochemistry assays were performed using anti-DOR (1/200) generated in our laboratory [14], anti-TP53INP1 (1/50, Santa Cruz) and anti-p62 (1/1000, BD). Hoechst (1/2000) (Molecular Probes, Oregon USA) was used to label DNA. Cells were mounted in Fluoromount (Electron Microscopy Sciences).

Immunofluorescence microscopy of cells was performed with Leica TCS SP5 and Leica TCS SPE (Leica Lasertechnik GmbH, Mannheim, Germany) confocal scanning microscopes adapted to an inverted LEICA DMI 6000CS and an upright LEICA DM 2500 microscope respectively. These instruments permitted the sequential acquisition of multiple cellular staining. Samples were scanned using a 63× oil objective and a zoom ranging from 1 to 3.5 to analyze intracellular regions using the LAS AF software from LEICA. The fluorochromes used (DAPI, GFP, Alexa-Fluor 561 and Alexa 647) were excited with 405 nm (UV), 488 nm, 561 nm and 633 nm laser lines respectively. To prevent bleed-through effects (i.e crosstalk of different fluorescence emission) in double or triple staining experiments, each dye was scanned independently. Moreover, by doing separate acquisitions and exciting in one wavelength (green 488 nm) and acquiring images in the higher one (red 561 nm), we controlled that the green channel does not emit in the red one. The projection format was 512×512 or 1.024×1.024. Images were acquired from 10 to 20 optical sections depending on the cell type analyzed. Figures were assembled from the TIFF and LIF files using open software ImageJ (author: Wayne Rasband, National Institute of Mental Health, Bethesda, Maryland, USA). PDM images were calculated using Image J “ICA plugin” (Tony Collins, McMaster University, Ontario), as previously described (8).

### Western blot analyses

Proteins from total homogenates were resolved in 10% or 15% acrylamide gels for SDS-PAGE and transferred to Immobilon sheets. Membranes were incubated with the following antibodies: anti-DOR (1/200) generated in our laboratory [14], anti-TP53INP1 (1/100, Santa Cruz), anti-p62 (1/1000, BD), anti-LC3 (1/1000, MBL), anti-beta-actin antibody (1/10000, Sigma) or anti-tubulin (1/8000, Sigma). ECL detection was performed as described [23].

### Real-time PCR

Total RNA extraction and treatment with DNase I were performed with Rneasy mini kit (Qiagen). RNA concentration was determined by spectrophotometry at an absorbance of 260 nm. Real-time PCR was performed from 0.1 µg of total RNA from HeLa cells using Taqman Probe for TP53INP1. Beta-actin mRNA was assayed as control.

### GST pull-down assays

GST protein was expressed in *Escherichia coli* (*E. coli*) strain LE392 and GST-fusion proteins were expressed in *E. coli* strain BL21 (DE3) cells (Invitrogen). GST and GST-fusion proteins were purified and immobilized on glutathione-coupled sepharose beads (Glutathione-Sepharose 4 Fast Flow, Amersham Bioscience). *In vitro* translations of GFP-tagged proteins were done using a coupled transcription-translation system (TNT, Promega Corp., Madison, WI) in the presence of [^35^S] methionine (Amersham Biosciences), following the manufacturer's instructions. GST pull-down assays were performed by incubating GST and GST-fusion proteins with the proteins translated *in vitro*. Briefly, the ^35^S-labeled proteins translated *in vitro* were diluted 20× with NET-N buffer (20 mM Tris-HCl, pH 8.0, 100 mM NaCl, 1 mM EDTA, 0.5% Nonidet P-40) containing Complete Mini EDTA-free protease inhibitor cocktail. The diluted products were pre-cleared by incubating with glutathione-coupled sepharose beads for 30 min prior to incubation with purified GST or GST-fusion proteins for at least 2 h at 4°C with gentle agitation. Unbound proteins were removed by washing the resins five times with NET-N buffer. The proteins were then eluted by boiling for 5 min in the presence of 2×-SDS gel loading buffer and then separated by SDS-PAGE. After vacuum drying the gel, radioactive signals were collected by autoradiography.

### Peptides

Peptides corresponding to human DOR were prepared using standard Fmoc solid-phase peptide synthesis with 0.1 mmol FastMoc protocols, HBTU/HOBt or HATU coupling using the Fmoc-Rink amide (Novabiochem) or Rink amide coupled ChemMatrix® (Matrix Innovation) resins. Peptides were prepared either manually in polypropylene syringes each fitted with a polyethylene porous disk or using an Applied Biosystems peptide synthesizer. All peptides had the N-terminal amino group acetylated and an amide at the C-terminus to prevent the presence of artificial charges at the two termini. Peptides were purified by reverse-phase chromatography and analyzed by MALDI-TOF mass spectrometry. Neutralized peptide solutions were kept as aliquots at −20°C.

### NMR spectroscopy

All experiments were performed at 12°C either at 90% H_2_O/10% D_2_O or at 100% D_2_O at pH6.5, on a Bruker Avance III 600-MHz spectrometer equipped with a z pulse field gradient unit and a triple resonance probe head. All peptides were assigned using 2D homonuclear NMR spectroscopy (Sattler et al., 1999). 2D TOCSY experiments used 35 and 60 ms mixing times while NOESY experiments used 250 and 125 ms mixing times.

### Statistical analysis

The data presented here were analyzed by t-test for unpaired groups or analysis of variance (ANOVA) and further post-hoc t-test.

## Results

### Identification of the *DOR/Tp53inp2* gene family

**Figure 1:**
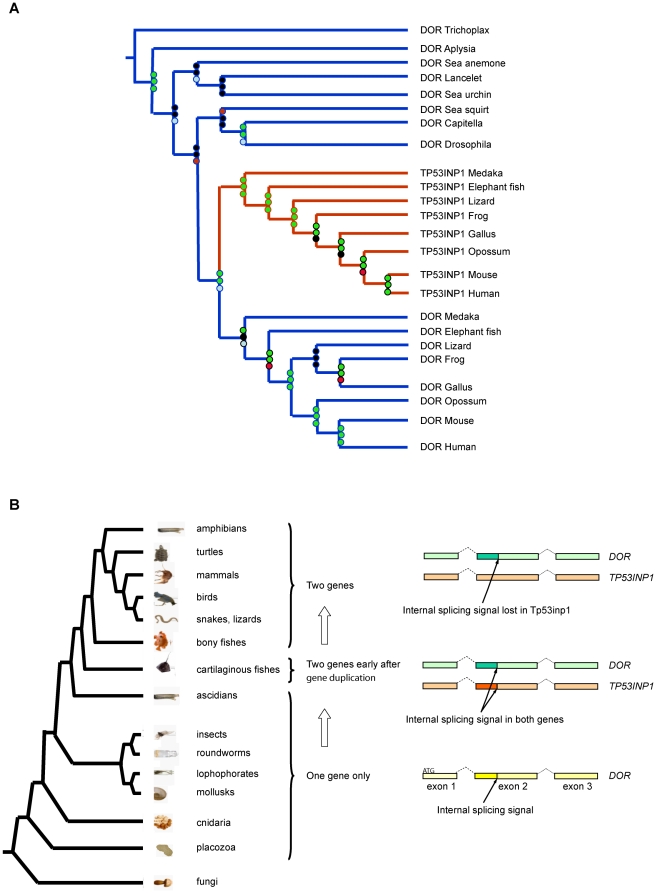
Identification of the *DOR* gene family. Panel A. Phylogenetic tree showing the two well supported clusters corresponding to DOR and Tp53inp1 vertebrate sequences and indicating a duplication of the ancestral *DOR* gene in the common ancestor of vertebrates around 450–510 Mya [38]. The Maximum Likelihood tree was generated using the WAG model in RaxML. Jackknife and bootstrap values were computed and are shown on each node. The top circle represents the Jackknife Support value using the Gonnett scoring matrix in parsimony. The middle circle at each node represents the Jackknife Support value with all characters set as unordered. The bottom circle refers to the bootstrap support computed using likelihood (WAG model). The final ML Optimization Likelihood was −10910.664029. Panel B. Diagram depicting the absence of an alternative exon 2 internal splicing signal in *Tp53inp1* genes. Exons shown correspond to the coding exons of the human genes. An alternative splicing signal present in exon 2 of *DOR* and absent in *Tp53inp1* genes from bony fish to mammals suggests *DOR* is the ancestral gene. The phylogenetic tree is based on our recent understanding of relationships of major taxonomic groups.

To retrieve as many sequences belonging to the DOR gene family as possible, we performed blast searches using known sequences of DOR/TP53INP2 and TP53INP1 (TP53INP1 is a DOR homologous protein also named SIP, Teap or Stinp, [14], [24], [25], [26] in the NCBI ESTs and protein databases). This approach yielded DOR or TP53INP1-like sequences only in metazoan organisms. For our phylogenetic and comparative analyses, we selected only sequences from representative metazoan organisms with sequenced genomes in which we could determine their intron/exon gene structure. Phylogenetic analyses revealed two well supported clusters corresponding to DOR and TP53INP1 sequences in vertebrates spanning from cartilaginous and bony fish to mammals (Figure 1A). These DOR and TP53INP1 protein clusters have probably branched out in the common ancestor of vertebrates since, despite thorough searches, we found only a single *DOR* or *Tp53inp1*-related gene in genomes of organisms older than cartilaginous fish (Figure 1 and Table S1).

To further characterize this protein family and to establish which of the two genes, *DOR/Tp53inp2* or *Tp53inp1*, is the ancestral gene, we analyzed the synteny conservation between *DOR/Tp53inp2* and *Tp53inp1* genomic locus and the locus of the single related genes in *Ciona intestinalis* (sea squirt), *Strongylocentrotus purpuratus* (sea urchin) and *Drosophila* (fruit fly). We observed synteny conservation for *DOR/Tp53inp2* and Tp53inp1 genomic regions from mammals to bony fish, but it was lost in sea squirt, sea urchin and fruit fly (data not shown). Although the synteny analysis was not conclusive, the presence of an alternative splice signal in all *DOR/Tp53inp2* genes from from *Callorhinchus milii* (elephant fish) to human as well as in some of the ancient single related genes and the absence of this splicing signal in *Tp53inp1* genes (except for *Tp53inp1* from elephant fish) supports *DOR/Tp53inp2* as the ancestral gene. While the intron/exon gene structure is conserved between all DOR family members (*DOR/Tp53inp2*, *Tp53inp1* and single related genes, Figure S1), only *DOR/Tp53inp2* and single related genes from sea squirt, *Capitella sp* (annelids), fruit fly, *Nematostella vectensis* (sea anemone) and *Trichoplax adhaerens* (placozoa), have an internal splicing signal in exon 2 (an intron in sea squirt) which produces a short isoform that is absent in all *Tp53inp1* genes (except for elephant fish *Tp53inp1*; Figure 1B, Figure S1 and Table S1). These data support the view that *DOR/Tp53inp2* is the ancient gene. The finding of this splice signal in the *Tp53inp1* gene in cartilaginous fish (elephant fish), which is around 50 My older than bony fish, in addition to the size similarity (215 amino acids long) between elephant fish TP53INP1 and DOR/TP53INP2 proteins suggest that elephant fish *Tp53inp1* is close to the gene duplication event.

**Figure 2:**
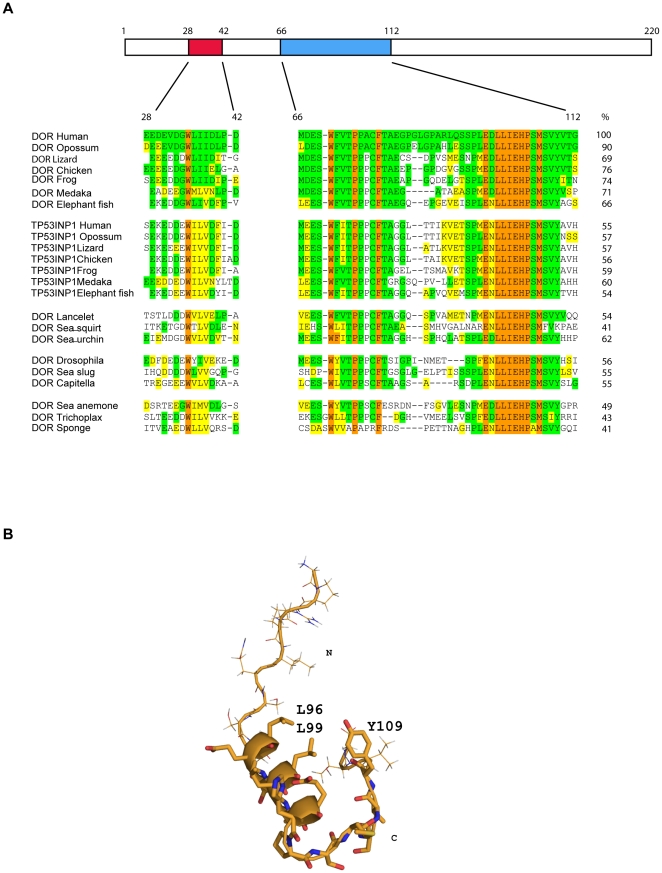
Multiple alignment of two protein regions. Secondary structure of region 2. Panel A. Alignment of two DOR and TP53INP1 protein regions conserved from sponges to mammals. The alignment is shaded with respect to the human DOR protein: orange, identical in all sequences; green, identical; and yellow similar to human DOR. Residue numbers correspond to human DOR protein. Identities of regions 1 and 2 (integrated) compared to human DOR are shown as percentages on the right. Panel B. Secondary structure of region 2. The helical part of the peptide is shown as a ribbon representation with some side chains highlighted. The structure shown corresponds to that of the lowest energy after water refinement from an ensemble of 80 structures. The structures were calculated using a simulated annealing protocol using NOEs and J-couplings as restraints.

The overall sequence identity between all DOR proteins (from elephant fish to humans) is 48.5±11.6%, (Figure S1 and Table S2). Conservation of all TP53INP1 proteins is 53.7±3.7% (identity), and lower when all DOR and TP53INP1 protein sequences were compared (32.9±4.0% identity). Interestingly, two well-conserved regions are evident in all sequences of DOR gene family members from human to basal metazoans (placozoa and sponge) (Figure 2A and Figure S1). In human DOR, these regions localize to positions 28–42 (region 1) and 66–112 (region 2) (Figure 2A). These regions are unique to the DOR protein family and do not show identity with any other protein sequences in databases. When comparing these DOR regions in human and in placozoa (*Trichoplax adhaerens*) globally, they showed 43% identity (Figure 2A). Human DOR and TP53INP1 display 55% identity in these regions (Figure 1B). Furthermore, a nuclear export sequence (NES) motif has previously been identified in region 1 (in residues 32 to 40) of DOR protein [12]. Region 1 is short, comprising about 15 residues, half of which are negatively charged. The second conserved region contains 56 residues and has a highly hydrophobic C-terminal region. We hypothesized that the conservation of these segments between highly divergent regions is linked to maintaining some secondary/tertiary structure required for the protein function. To test this hypothesis, we ran secondary structure predictions on the regions using the PSIPRED server [27]. According to the prediction, the first motif does not have a defined tendency to populate a given secondary structure. In contrast, the second motif has a medium to high tendency to populate the helical conformation in the region from L96 to H103 (confidence values of 8–9). To determine whether this prediction can be experimentally detected, we synthesized several peptides spanning the region from G87 to G112 (numbering corresponds to human DOR) and studied the structural properties using homonuclear Nuclear Magnetic Resonance in solution. Using sets of 2D TOCSY and NOESY experiments run with a range of mixing times, we detected a pattern of NOEs compatible with helical properties for the central part of the peptide sequence, in good agreement with the prediction. In addition, the first nine residues did not show contacts with the rest of the sequence but could be unambiguously assigned and sequentially connected, thereby corroborating the prediction of flexibility. With respect to the last six residues, they adopted an extended but not totally flexible conformation. Indeed, we observed a few weak NOEs from the Y109 ring to I101 methyl groups, suggesting that, at least under our experimental conditions, some peptides have a close conformation in which the tyrosine ring shields the hydrophobic side of the helix from the solvent (Figure 2B).

### Different DOR/TP53INP2 and TP53INP1 proteins show transcriptional activity

**Figure 3:**
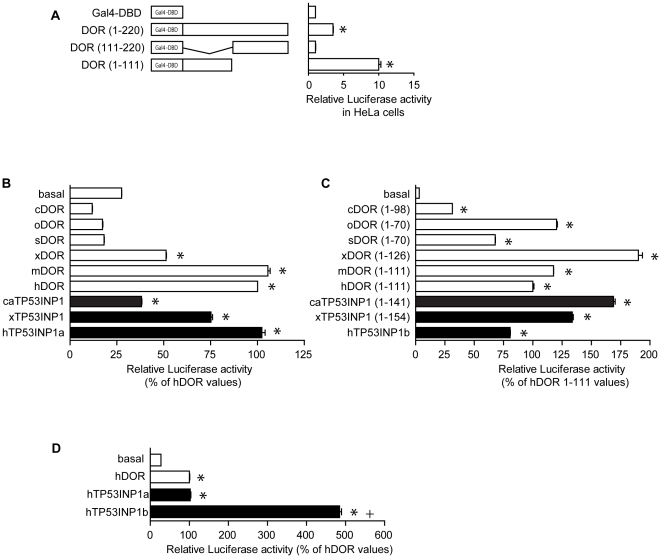
Transcriptional activity of various members of the *DOR* gene family and mutational analysis of human DOR. Panel A. Human DOR or fragments corresponding to the amino acids indicated were fused to the DNA-binding domain of Gal4 (Gal4 DBD) and transfected in HeLa cells. Transcription was assayed with a reporter plasmid containing five copies of the UAS linked to luciferase. Results are mean ± SEM of 6 independent experiments. * significant difference compared to the Gal4 DBD-DOR group, at P<0.0001. Panels B and C. DOR orthologs (panel B) or the N-terminal fragments of the DOR orthologs (panel C) were fused to the Gal4-DBD and transfected in HeLa cells. Transcription was assayed as in previous panels and values were expressed as % of human DOR values. cDOR, *Ciona intestinalis* DOR. oDOR, *Oncorhynchus mykiss* DOR. sDOR, *Salmo salar* DOR. xDOR, *Xenopus laevis* DOR. mDOR, *Mus musculus* DOR. hDOR, *Homo sapiens* DOR. caTP53INP1, *Callorhinchus milii* TP53INP1. xTP53INP1, *Xenopus laevis* TP53INP1. hTP53INP1, *Homo sapiens* TP53INP1. hTP53INP1a refers to isoform a (NP 150601, 240 aa), and hTP53INP1b refers to isoform b, generated by a transcript variant, which contains an alternate exon that has an in-frame stop codon in it, and therefore has a shorter and distinct C-terminus compared to isoform a (NP 001129205, 164 aa). Results are mean ± SEM of triplicates and are representative of 3 independent experiments. * significant difference compared to the basal group, at P<0.0001. Panel D. Transcriptional activity of the isoforms a and b of human TP53INP1. Values were expressed as % of human DOR values. Results are mean ± SEM of triplicates and are representative of 3 independent experiments. * significant difference compared to the basal group, at P<0.0001; ^+^ significant difference compared to the isoform a of Tp53inp1, at P<0.0001.

DOR/TP53INP2 activates thyroid hormone receptors and ecdysone receptors in mammalian cells and in Drosophila, respectively [14], [15]. In addition, TP53INP1 modulates the activity of p53 and p73 transcription factors [26], [28], [29]. In this regard, full-length human DOR or distinct cDNA fragments were fused to a Gal4 DNA-binding domain (Gal4-DBD) and transcriptional activity was assayed by co-transfection with a Gal4 reporter plasmid in HeLa cells. Gal4-DBD fused to full-length DOR caused a moderate increase in reporter activity (Figure 3A) and deletion of the C-terminal half of the protein (fragment 1–111) markedly increased this activity (9-fold) (Figure 3A). The C-terminal half of DOR (111–220) showed no transcriptional activity (Figure 3A). Thus, we next studied whether different DOR or TP53INP1 proteins from a variety of chordate species (ranging from prochordates with only one gene, elephant fish with two genes close to the duplication event or mammals) also show transcriptional activity when tested in human cells. To this end, full-length DOR or TP53INP1 or cDNA fragments corresponding to the 1–111 fragment of human DOR were fused to Gal4 DNA-binding domain (Gal4-DBD) and transcriptional activity was assayed by co-transfection with a Gal4 reporter plasmid in HeLa cells. The cDNAs selected were *Ciona intestinalis* (sea squirt) DOR, *Oncorhynchus mykiss* DOR (rainbow trout, 29% identical to human DOR), *Salmo salar* DOR (salmon, 28% identical to human DOR), *Xenopus laevis* DOR (frog, 29% identical to human DOR), *Xenopus laevis* TP53INP1, *Callorhinchus milii* (elephant fish) TP53INP1, mouse DOR, and human TP53INP1.

Analysis of the full-length cDNAs fused to Gal4-DBD indicated a potent transcriptional activity driven by frog DOR and TP53INP1, mouse DOR and human DOR and TP53INP1 (Figure 3B). Further study of the N-terminal halves of the proteins revealed transcriptional activity driven by all the DOR and TP53INP1 proteins studied, including sea squirt DOR and elephant fish TP53INP1 (Figure 3C). We have also analyzed the relative potency of the full-length human TP53INP1 (isoform a or SIP27, http://www.ncbi.nlm.nih.gov/gene/94241) (240 amino acid residues), and the short TP53INP1 isoform b (or SIP18, 164 amino acid residues from which the first 157 residues are shared with the long form) after fusion to Gal4-DBD. Under our experimental conditions, the human TP53INP1 isoform b was more active than the long isoform a (Figure 3D).

### Autophagic activity of other members of the *DOR/Tp53inp2* gene family: human *TP53INP1* modulates autophagy

**Figure 4:**
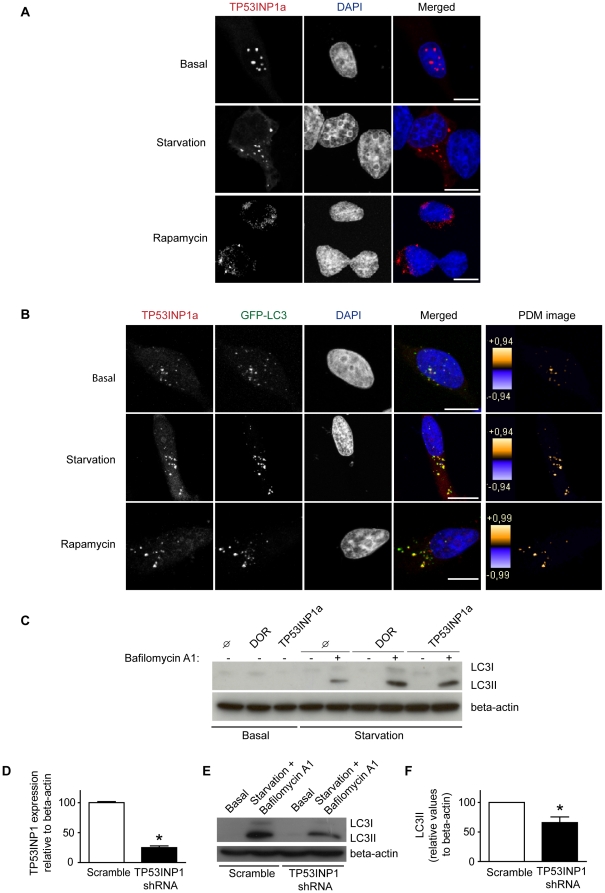
Human TP53INP1 activates autophagy. Panel A. HeLa cells were transiently transfected with TP53INP1 and incubated with DMEM (basal) or HBSS for 1 h (amino acid starvation), or 2 µM rapamycin for 3 h. The intracellular localization of TP53INP1 was analyzed by immunofluorescence and is shown in red. The nuclei are shown in blue. Scale bars, 10 µm. Panel B. Confocal images of HeLa cells transiently transfected with TP53INP1 and GFP-LC3, and incubated whether with DMEM, HBSS for 1 h or 2 µM rapamycin for 3 h. The intracellular localization of TP53INP1 was analyzed as in panel A. Scale bars, 10 µm. Contrast-corrected merged RGB pictures and Z-projection of PDM images are shown for all panels. Color scales of PDM images have different maximal values so care must be taken when comparing conditions. PDM values closer to 1 show reliable co-localized pixels. Panel C. HeLa cells were transiently transfected with either pcDNA3, DOR or TP53INP1a and incubated with DMEM or HBSS for 1 h, alone or in combination with 200 nM bafilomycin A1 for 16 h (cells were incubated with BafA1 for 16 h and for the last hour they were incubated with or without HBSS). Cell lysates were obtained and Western blot assays were performed with specific antibodies against L C3 and beta-actin. Panel D. HeLa cells previously infected with lentiviruses encoding scramble RNA or TP53INP1 shRNA were cultured. Cell extracts and total RNA were obtained and TP53INP1 mRNA levels were assayed by real-time PCR. Data are mean ± SEM of 3 independent experiments. * significant difference compared to the Scramble group, at P<0.0001. Panels E and F. Scramble or TP53INP1 shRNA HeLa cells were cultured and incubated with DMEM or HBSS for 1 h in combination with 200 nM bafilomycin A1 for 16 h (cells were incubated with BafA1 for 16 h and for the last hour they were incubated with or without HBSS). Endogenous LC3 and beta-actin were detected by Western blot (panel E). Quantification of LC3II levels relative to beta-actin was performed (panel F). Data are mean ± SEM of 3 independent experiments. * significant effects caused by TP53INP1 knockdown, at P<0.05.

We next analyzed whether the other *DOR* gene family member, *TP53INP1*, also regulates autophagy. To this end, HeLa cells were transfected with vectors encoding human TP53INP1 (isoform a). Activation of autophagy by amino acid starvation or by rapamycin treatment of TP53INP1-transfected cells caused these proteins to leave the nucleus and to re-localize to cytoplasmic punctate structures similarly to what DOR does (Figure 4A). We next analyzed whether TP53INP1 co-localizes with autophagosomes. To this end, HeLa cells were co-transfected with GFP-LC3 and TP53INP1, and immunofluorescence microscopy was performed with cells subjected to a range of conditions. Under basal conditions, TP53INP1 isoform a was detected both in the nucleus and in the cytosol, and it co-localized with GFP-LC3 in both locations. Of note, TP53INP1 is detected mainly in the nucleus, but part of the protein is found in the cytosol due to GFP-LC3 co-transfection, similarly to what detected for DOR [12]. During starvation, TP53INP1 and GFP-LC3 were found in cytosolic punctate structures (Figure 4B). Image analysis indicated a substantial co-localization of TP53INP1 and GFP-LC3 in starvation conditions and in the presence of rapamycin, similar to the co-localization of DOR and GFP-LC3 [12].

Furthermore, overexpression of TP53INP1 or DOR enhanced the autophagic flux in HeLa cells. When lysosomal degradation was inhibited using bafilomycin A1, induction of autophagy caused an increase in LC3-II abundance in TP53INP1- or DOR-transfected cells as compared with control cells (Figure 4C). In order to provide additional evidence for the role of TP53INP1 in autophagy, HeLa cells were infected with lentiviruses encoding shRNA directed against *TP53INP1* or with scrambled shRNA-expressing lentiviruses. shRNA-infected cells showed a marked *TP53INP1* repression (80% reduction) (Figure 4D). In keeping with a role of TP53INP1 in autophagy, silencing of *TP53INP1* caused a reduced accumulation of LC3-II in response to amino acid starvation and to bafilomycin A1 blockade of lysosomal activity (Figures 4E and F). In all, our data indicate that human TP53INP1, similarly to human DOR protein, is an activator of autophagy.

### DOR regions 1 and 2 contain signals that are crucial for transcriptional activity

**Figure 5:**
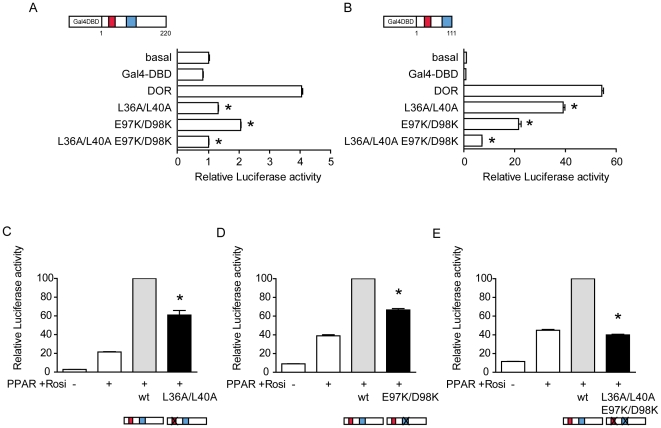
Effects of DOR mutants L36A/L40A or E97K/D98K on transcriptional activity. Panels A and B. DOR, two mutated versions of DOR and a combination of the two mutants in the full-length protein (panel A) or in the N-terminal portion (panel B) were transfected in HeLa cells. Transcription was assayed as in previous figure. Results are mean ± SEM of triplicates and are representative of 3 independent experiments. * significant difference compared to the wild-type group, at P<0.0001. Panels C, D and E. HeLa cells were transfected with the expression plasmid encoding PPARγ, the reporter vector containing PPARγ response elements linked to luciferase, wild-type DOR and either the mutant L36A/L40A (panel C) or the mutant E98K/D99K (panel D) or the quadruple mutant L36A/L40A E98K/D99K (panel E). Cells were treated for 16 hours in the presence of 100 nM rosiglitazone and assayed for reporter expression. Results are mean ± SEM of triplicates and are representative of 3 independent experiments. * significant difference compared to the wild-type group, at P<0.05.

On the basis of the identification of conserved regions 1 and 2 in the DOR gene family, we next studied their relevance in transcriptional activity. Regions 1 and 2 are detected in the N-half of human DOR protein, which contains the potential transcriptional activity when assayed in vitro (Figure 3A). To this end, we studied the effect of DOR mutants. In region 1, we generated the human DOR mutant L36A/L40A, positions in which hydrophobic and branched-chain amino acids are conserved through evolution, and which are part of a nuclear export signal [12]. In region 2 we generated the DOR mutant E97K/D98K, since these residues are conserved between Trichoplax and human DOR (Figure 2A). In initial studies, the full-length human wild-type DOR or mutant forms were fused to a Gal4 DNA-binding domain (Gal4-DBD) and transcriptional activity was assayed by co-transfection with a Gal4 reporter plasmid in HeLa and in HEK293T cells. Data indicated that the mutant L36A/L40A (region 1) showed a lower transcriptional activity compared to the wild-type DOR (Figure 5A, B). The DOR mutant form E97K/D98K (region 2) also showed less transcriptional activity than the wild-type (Figure 5A, B), and the mutant L36A/L40A/E97K/D98K (regions 1 and 2) showed negligible transcriptional activity (Figure 5A, B).

**Figure 6:**
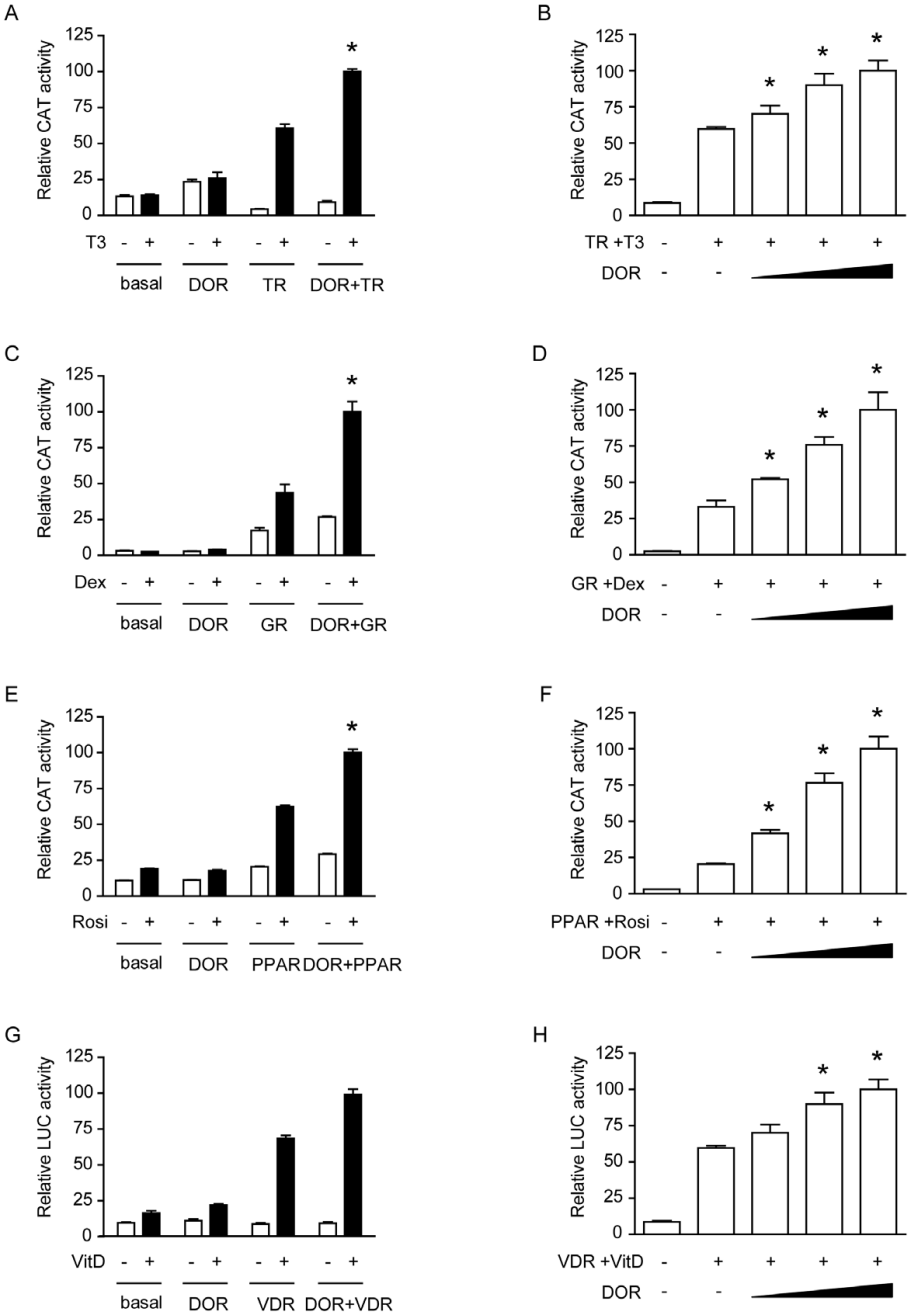
DOR transactivates nuclear hormone receptors. Panels A, C, E and G. HeLa cells were transfected with expression plasmids encoding TR_α1_, GR, PPAR_γ_ or VDR, DOR, the empty vector pcDNA3 as a control vector and the reporter vectors containing TR_α1_, GR, PPAR_γ_ or VDR response elements linked to CAT or luciferase. Cells were treated for 18 h in the presence or absence of ligands (100 nM T_3_, 100 nM dexamethasone, 100 nM rosiglitazone or 10 nM 1,25-dihydroxi-vitamin D) and assayed for reporter expression. Results are mean ± SEM of triplicates and are representative of 6 independent experiments. * significant difference compared to the nuclear hormone receptor group, at P<0.05. Panels B, D, F and H. Reporter assays were done as in previous panels but different amounts of DOR (ranging from 200 to 600 ng) were used for transfection and in the presence of ligands. Results are mean ± SEM of triplicates and are representative of 6 independent experiments. * significant difference compared to the nuclear hormone receptor group, at P<0.05.

**Figure 7:**
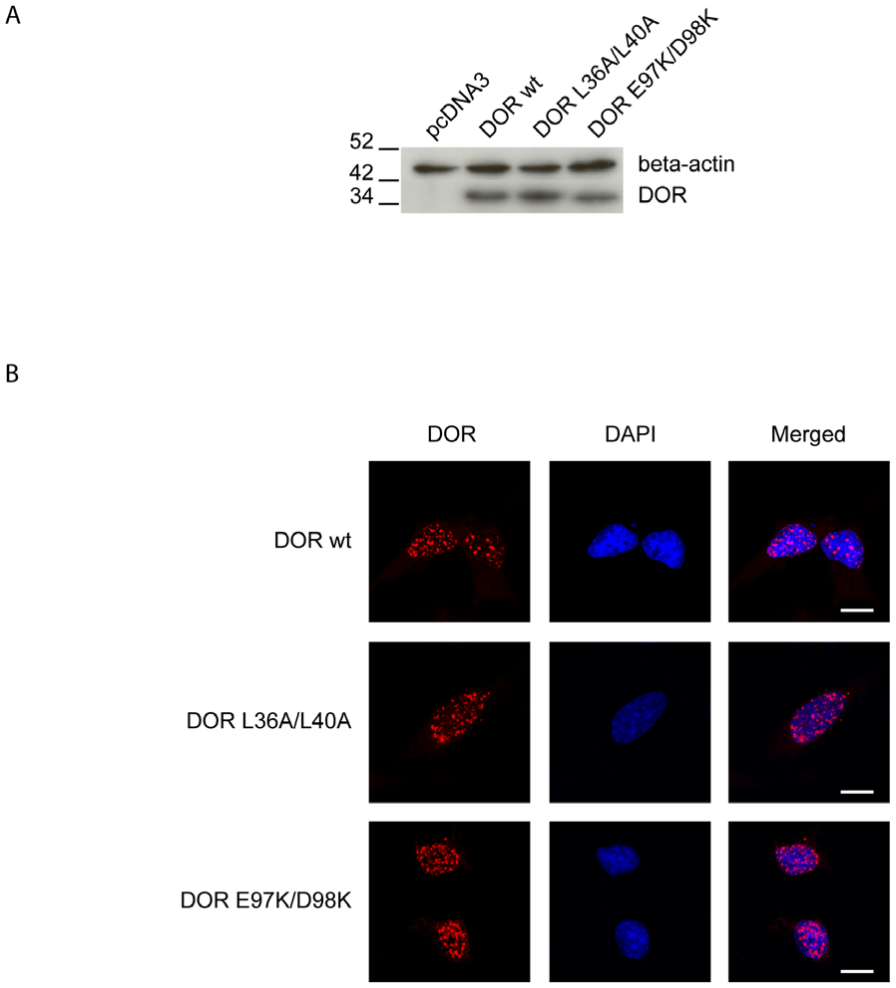
DOR mutants L36A/L40A or E97K/D98K localize in the nucleus. Panel A. Expression of wild-type and mutant forms L36A/L40A or E97K/D98K of DOR. Western blot assays were performed in cell extracts by using specific antibodies against DOR and beta-actin. Panel B. Intracellular distribution of DOR mutants. HeLa cells were transiently transfected with wild-type or mutant forms L36A/L40A or E97K/D98K of DOR. The intracellular localization of DOR was analyzed by immunofluorescence and is shown in red. The nuclei are shown in blue (DAPI staining). Scale bars, 10 µm.

The co-transfection of DOR and nuclear hormone receptors (TRα1, GR, PPARγ, or VDR) enhanced the transcriptional activity of the reporter gene in a dose-dependent manner and only in the presence of the ligand (Figure 6). These effects were specific for DOR overexpression, and transfection with a plasmid encoding the xCT amino acid transporter did not cause any effect (data not shown). DOR did not affect the reporter activity induced by transcription factors p53 or c-Myc (data not shown), indicating that DOR does not act as a general co-activator of any transcription factor. Mutant forms of DOR in region 1 or region 2 were also tested for co-regulator activity by co-transfection with PPARγ in HeLa cells. The L36A/L40A and E97K/D98K mutants showed less transactivation activity than the wild-type DOR (Figure 5C, D) under conditions in which both mutant forms were equally overexpressed in cells (Figure 7A). The mutant L36A/L40A/E97K/D98K lost all transactivation activity (Figure 5E).

### DOR regions 1 and 2 contain signals that are crucial for autophagic activity

**Figure 8:**
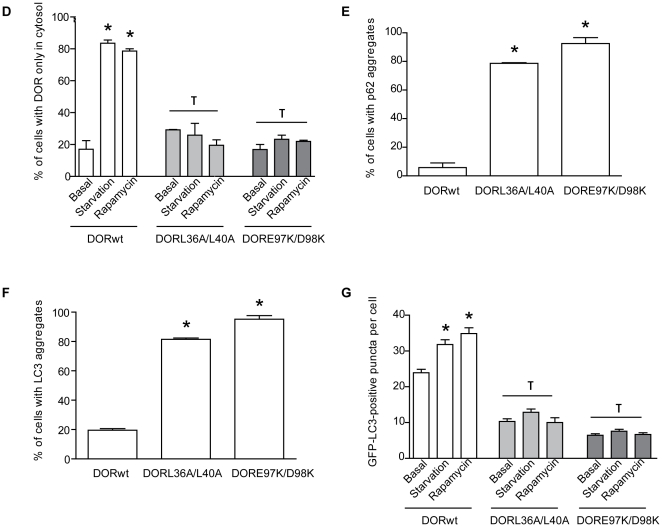
Effects of DOR mutants L36A/L40A or E97K/D98K on autophagosome formation. Panels A, B and C. Confocal images of stable GFP-LC3 HeLa cells transiently transfected with either wild-type DOR (panel A), L36A/L40A (panel B) or E97K/D98K (panel C). HeLa cells were incubated with DMEM or HBSS for 1 h (amino acid starvation) or 2 µM rapamycin for 3 h. Endogenous p62 was labeled in red and DOR was labeled in cyan. Nuclei were stained with DAPI. Scale bars, 10 µm. Panel D. Image analysis on the intracellular distribution of DOR in HeLa cells subjected to activation of autophagy. DOR intracellular localization was analyzed by immunofluorescence in a minimum of 100 randomly chosen transfected HeLa cells in each experiment. Cells were categorized as having DOR exclusively located in the cytosol or also in nucleus. Data are mean ± SEM of 4 independent experiments. * significant effects of starvation and rapamycin treatment, at P<0.001; τ significant effects caused by DOR mutants overexpression, at P<0.001. Panels E and F. Image analysis on the abundance of endogenous p62-positive puncta and GFP-LC3 aggregates per cell was performed. Number of p62-positive puncta (panel E) and number of GFP-LC3 aggregates (panel F) were analyzed by immunofluorescence in a minimum of 100 randomly chosen transfected HeLa cells in each experiment. Data are mean ± SEM of 4 independent experiments. * significant effects caused by DOR mutants overexpression, at P<0.001. Panel G. Image analysis on the abundance of GFP-LC3 puncta. GFP-LC3-positive vacuoles were counted in 100 transfected HeLa cells. Data are mean ± SEM of 4 independent experiments. * significant effects of starvation, at P<0.001; τ significant effects caused by DOR mutants overexpression, at P<0.001.

Next, we studied whether regions 1 and 2 in the DOR sequence contribute to the autophagic activity exerted by this protein in mammalian cells [12], [13] and in fruit flies [12]. In order to further demonstrate the involvement of regions 1 and 2 in autophagy, HeLa cells were transfected with vectors encoding wild-type or mutant versions of DOR. The overexpression of L36A/L40A or E97K/D98K DOR mutants was similar to the wild-type form (Figure 7A) and they localized in punctuated structures in the nucleus (Figure 7B). Neither wild-type DOR nor mutants L36A/L40A and E97K/D98K DOR were degraded through autophagy (data not shown). We next analyzed whether the mutant forms of DOR in regions 1 and 2 underwent translocation from the nucleus to the cytosol in response to autophagy activation, and whether they co-localized with autophagosomes. To this end, stable GFP-LC3 HeLa cells were transfected with either wild-type DOR or mutant forms, and immunofluorescence microscopy was performed with cells subjected to a range of conditions. Under basal conditions, wild-type DOR was detected mainly in the nucleus, while GFP-LC3 and p62 were found mainly in the cytosol. During stimulated autophagy, wild-type DOR and GFP-LC3 co-localized in cytosolic punctuate structures (Figure 8A and D). Under autophagy-activated conditions, p62 substantially co-localized with GFP-LC3 (Figure 8A). No significant co-localization was found between DOR and p62. In contrast, L36A/L40A DOR mutant showed a number of differences to the wild-type form. Under basal conditions, when L36A/L40A was mainly in the nucleus, we detected an increase in the percentage of cells with p62- and LC3-positive cytosolic aggregates (Figure 8E, and F). Moreover, amino acid starvation and rapamycin caused only a minimal exit of L36A/L40A DOR from the nucleus (Figure 8B, and D).

The E97K/D98K DOR mutant showed a similar pattern to that of the L36A/L40A mutant. Under basal conditions, although the nuclear distribution of E97K/D98K DOR was comparable to the wild-type form, we detected an increase in the percentage of cells with cytosolic GFP-LC3-positive aggregates (Figure 8C, E, and F). Amino acid starvation caused incomplete exit of E97K/D98K DOR from the nucleus although the co-localization with GFP-LC3 in the cytosol was conserved (Figure 8C, and D). Of note, overexpression of DOR mutants L36A/L40A or E97K/D98K prevented proper autophagosome formation in response to starvation or rapamycin (Figure 8G).

Taken together, these results indicate that mutations in regions 1 or 2 alter the normal function of DOR in autophagy, and that their overexpression in HeLa cells inhibits the autophagic machinery. Moreover, these alterations are detectable under basal non-stimulated conditions.

### Identification of a functional LIR motif in region 1 of DOR and TP53INP1 proteins

**Figure 9:**
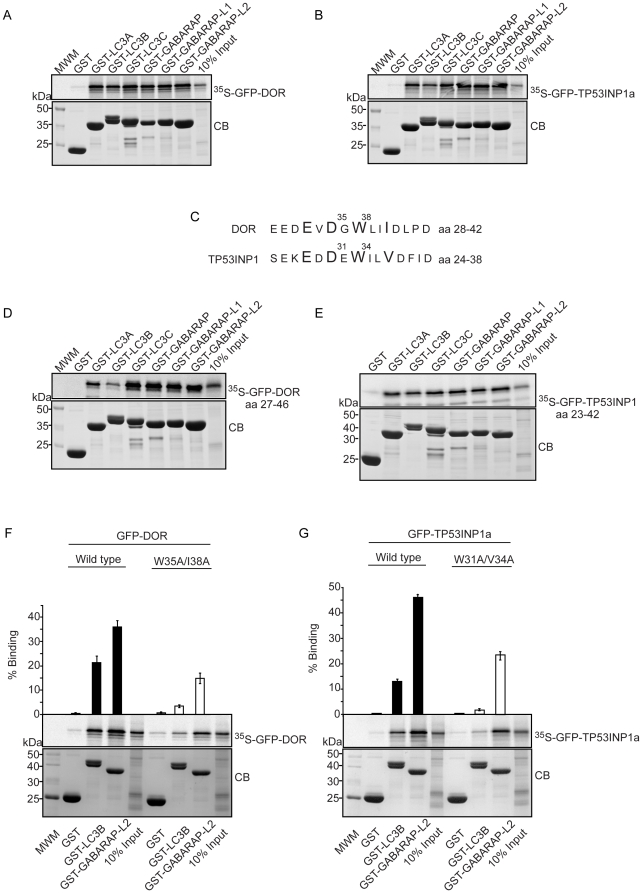
Identification of a LIR motif in region 1 of DOR and TP53INP1 proteins. Panels A and B. DOR and TP53INP1a interact with all human LC3/GABARAP family members. Full-length DOR (A) and TP53INP1 (B), both fused to GFP, were *in vitro* translated in the presence of [^35^S]methionine, and tested in GST pull-down assays for interaction with GST or the indicated GST fusion proteins. Panel C. Alignment of region 1 of human DOR and TP53INP1with the key conserved aromatic, hydrophobic and acidic residues for binding to LC3/GABARAP family proteins shown in larger fonts. Panels D and E. The putative LIR motifs of DOR (D) and TP53INP1 (E), both fused to GFP, were *in vitro* translated and tested in GST pull-down assays for interaction with GST or the indicated GST fusion proteins. Panels F and G. GST pull-down assays showing the effect of point mutations within the LIR motifs of DOR and TP53INP1a. Wild-type or mutated DOR (A) and TP53INP1a (B) fused to GFP were translated *in vitro* and tested for interaction with GST-LC3B or GST-GABARAP-L2. Quantifications of the mean % binding with standard deviations from three independent experiments are shown above the autoradiographs.

**Figure 10:**
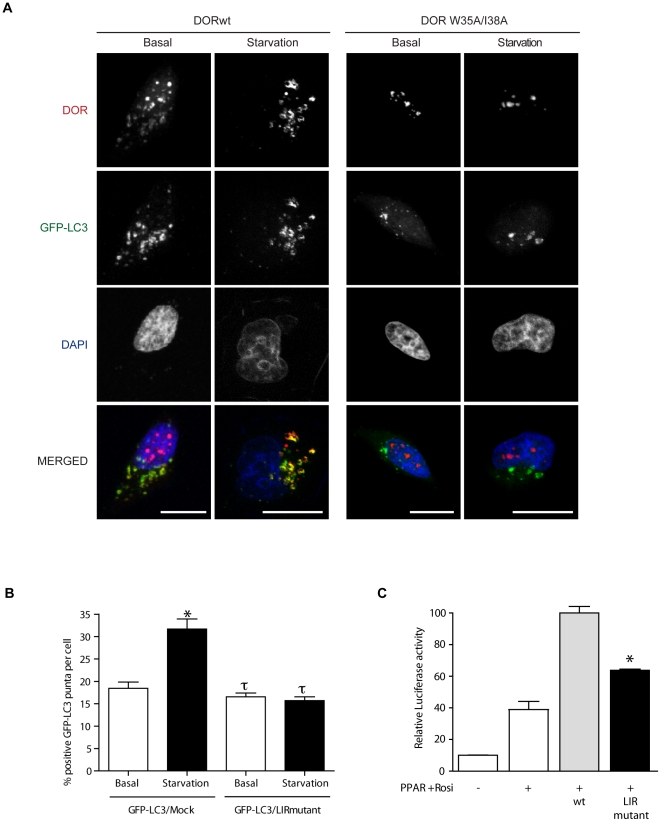
DOR mutant W35A/I38A does not exit from the nucleus during the activation of autophagy. Panel A. Confocal images of stable GFP-LC3 HeLa cells transiently transfected with either wild-type DOR (left panel) or W35A/I38A (right panel). HeLa cells were incubated with DMEM or HBSS for 1 h (amino acid starvation). DOR was labeled in red. Nuclei were stained with DAPI. Scale bars, 10 µm. Panel B. GFP-LC3-positive vacuoles were counted in 100 transfected HeLa cells (either transfected with GFP-LC3 and an irrelevant protein (TRα1) or with GFP-LC3 and DOR LIR mutant) in 3 independent experiments. * indicates significant effects of amino acid starvation, p<0.001; τ indicates significant effects caused by overexpression of DOR LIR mutant, p<0.001. Panel C. HeLa cells were transfected with the expression plasmid encoding PPARγ, the reporter vector containing PPARγ response elements linked to luciferase, wild-type DOR or the LIR mutant. Cells were treated for 16 hours in the presence of 100 nM rosiglitazone and assayed for reporter expression. Results are mean ± SEM of triplicates and are representative of 3 independent experiments. * significant difference compared to the wild-type group, at P<0.05.

Using GST pull-down assays, we found that DOR and TP53INP1 bound strongly to all six members of the human LC3/GABARAP protein family tested (Figure 9A and B). LIR (LC3 interacting region) motifs [20] mediate a specific interaction between selective autophagic substrates and cargo receptors, such as p62, NBR1 (neighbor of BRCA1 gene 1) and Nix and the autophagosome-associated LC3/GABARAP family proteins, and between yeast Atg19, Atg32 and Atg8 [30], [31]. Inspection of the amino acid sequences of DOR and TP53INP1 revealed that region 1 in both proteins harbors a typical LIR motif (Figure 9C). These motifs contain the aromatic (W) and hydrophobic (L/I) residues required for binding to the two hydrophobic pockets on the LC3/GABARAP family proteins as well as acidic residues N-terminal to the conserved aromatic residue that may interact with basic residue(s) in the N-terminal arm of the LC3/GABARAP family of proteins [31], [32]. When tested by GST pull-down assays peptide sequences spanning 20 amino acids containing the LIR motifs of DOR and TP53INP1 bound to the LC3/GABARAP proteins (Figure 9D and E). The DOR LIR peptide bound more weakly to LC3A and –B than to the other LC3/GABARAP family proteins. To characterize the LIR motifs further, we mutated the conserved aromatic (W) and hydrophobic residues (L/I) to alanines and tested the mutants in pull-down assays with GST-LC3B and GST-GABARAP-L2. For TP53INP1 and DOR, the mutations almost completely abolished binding to LC3B while the binding to GABARAP-L2 was reduced by about 50% (Figures 9F and G). This observation clearly demonstrates the relevance of these residues and confirms the identification of functional LIR motifs in region 1 of these two proteins. Regarding cell localization, DOR mutant W35A/I38A, like wild-type DOR, was nuclear under basal conditions. However, it lost its capacity to exit the nucleus under activation of autophagy (Figure 10A), similarly to the effects caused by mutation of the NES signal [12]. Furthermore, results indicated that overexpression of DOR LIR mutant caused a significant decrease of the GFP-LC3-positive puncta per cell under both basal and amino acid starvation conditions (p<0.001), exhibiting a dominant-negative effect on exogenous wild-type DOR function. In fact, overexpression of the LIR mutant prevented the cells to respond to autophagy activation by amino acid starvation (Figure 10B). In addition, DOR LIR mutant was also tested for co-regulator activity by co-transfection with PPARγ in HeLa cells. W35A/I38A mutations also impaired transcriptional activity of the protein (Figure 10C).

**Figure 11:**
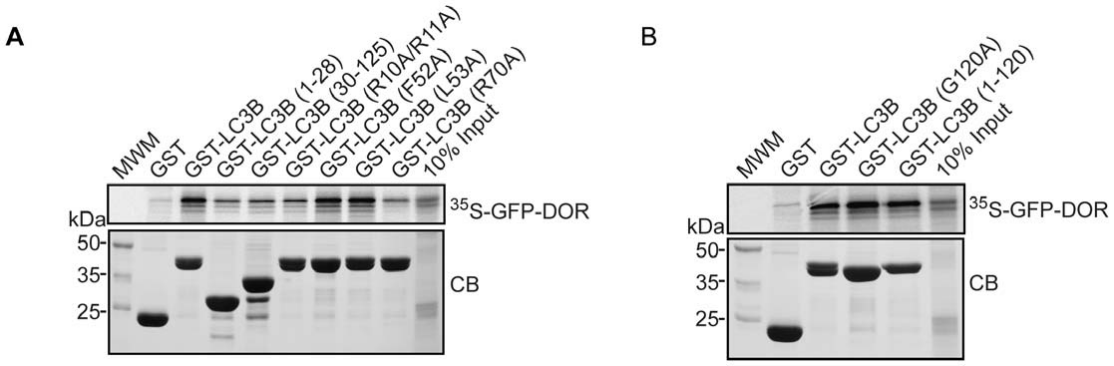
Mutant analyses of the interaction between LIR motifs in DOR and TP53INP1 and LC3/GABARAP family proteins. Panel A. GST pull-down assays showing the effect of deletions or point mutations in LC3B previously shown to affect LIR interactions. Panel B. DOR binds both to the pro-form and form I of LC3B. DOR fused to GFP was translated *in vitro* and tested for interaction with the indicated GST-LC3B constructs.

Both the N- and C-terminal domains of LC3B are required for the LIR-dependent interaction with p62, NBR1 and FYCO1 and point mutations in either domain affect this interaction [20], [21], [32], [33]. We found that the binding of DOR to LC3B required both domains of LC3B and the arginine residues (R10 and R11) of the N-terminal arm since the fragments GST-LC3B (1–28) and GST-LC3B (30–125) and the mutant GST-LC3B (R10A/R11A) showed reduced binding to DOR (Figure 11A). The latter residues possibly interact with the acidic residues N-terminal to the conserved W of the LIR motif. DOR bound equally well to full-length proform of LC3B as to form I LC3B (GST-LC3B(1–120)) (Figure 11B). Furthermore, DOR also bound to non-cleavable mutant full-length proform of LC3B (GST-LC3B (G120A)) (Figure 11B).

## Discussion

Here we describe the *DOR/Tp53inp2* gene family that is detected in all metazoan species studied, ranging from basal Trichoplax and sponges to humans. While vertebrates contain two genes, *DOR* and *Tp53inp1*, only one gene is present in the genomes of invertebrates. Indeed, vertebrate *DOR* and *Tp53inp1* members form two well-defined clusters in the phylogenetic tree reconstruction. Inspection of the intron/exon structure of the genes allowed us a to detect a conserved internal splicing signal in exon 2 present in the genes from lower metazoans to vertebrate *DOR/Tp53inp2* genes but absent in *Tp53inp1* genes. The molecular data available support the hypothesis that *DOR/Tp53inp2* is the ancestral gene and that *Tp53inp1* appeared by a gene duplication event in the common ancestor of vertebrates. Thus, our data complements the previous sequence analysis by Nowak et al [13] and expands the phylogenetic reconstructions to include distant metazoan species.

Given the unique property of human and mouse DOR/TP53INP2 as bifunctional proteins with autophagy- and transcription-related functions [12], [13], [14], [15], we analyzed whether other members of the DOR family also share these characteristics. Human TP53INP1 is a nuclear protein reported to regulate p53 function in response to cellular stress [26], [28], [29]. We therefore examined whether TP53INP1 also controls autophagy. Four key results demonstrate that TP53INP1 is a regulator of autophagy. First, TP53INP1 undergoes movement from the nucleus to the cytosol in response to rapamycin or amino acid starvation, both conditions that activate autophagy. Second, TP53INP1 is detected in autophagosomes under autophagic activation. Third, TP53INP1 enhances autophagic flux, as assessed by enhanced LC3-II. Fourth, TP53INP1 silencing reduces autophagic flux in HeLa cells. In addition, recent observations [34] indicating that TP53INP1^−/−^ MEF cells show higher basal levels of p62, which suggests impaired autophagy also support the results presented here.

We have also shown that TP53INP1 and other members of the DOR/Tp53inp2 gene family can in vitro modulate transcription through the N-terminal half of the molecule. Thus, sea squirt DOR, rainbow trout DOR, salmon DOR, frog DOR, frog TP53INP1, elephant fish TP53INP1, and human TP53INP1 showed transcriptional activity when fused to a Gal4-DNA binding domain, and deletion of the C-terminal half of the protein markedly increased their activity. This observation supports the notion that the transcriptional activity of the protein is subjected to intramolecular control. This is compatible with a model by which deletion of the C-terminal half of the protein induces a conformational change in the protein that leads to a more open structure resulting in a more active protein. The variable transcriptional activity levels shown by different proteins should be interpreted with caution because of the specific assay used. These assays involved the use of chimeric proteins and were performed in a human cell line (HEK293), which represents an artificial measure of the transcriptional activity of proteins from very distant species ranging from prochordates to mice. Overall, and on the basis of our results, we propose that DOR and TP53INP1 proteins are dual regulators of autophagy and of transcription in metazoan species.

The conservation of function shown by the members of the DOR gene family is not paralleled by strong sequence conservation. In fact, sequence conservation within the DOR gene family was low and essentially restricted to two regions, which in human DOR encompassed amino acid residues 28–42 (region 1) and 66–112 (region 2). Region 1 contains a NES consensus site Φ-x_3_-Φ-x-Φ-x-Φ (residues 32 to 40) (Φ = hydrophobic) [35], and its mutation (L36A/I37A/I38A/L40A) has been shown to retain DOR in the nucleus in response to rapamycin treatment [12]. Similarly, we found that mutation in positions 36 and 40 (L36A/L40A) partially prevented the exit of the protein to the cytosol in response to autophagy activation induced by rapamycin or amino acid starvation. In addition, the L36A/L40A DOR mutant caused an accumulation of p62, which suggests a reduced autophagic activity. Based on the observation that the double mutation of second and fourth Leu residues in the consensus sequence of SNUPN protein to Ala abolished CRM1-SNUPN interactions [36], we suggest that the DOR mutant L36A/L40A will show defective NES activity and therefore lack of CRM1 recognition.

In region 1, DOR also shows an LC3-interacting region (LIR), and mutation of the core LIR residues W35A/I38A blocks the nuclear exit in response to autophagy activation, and markedly reduces binding to LC3. This is in keeping with the observations that mutation of the hydrophobic Trp and Leu of the core LIR of p62 to Ala almost abolished the interaction with LC3 [32]. Thus, DOR gene family products add to the growing list of proteins, such as p62, NBR1 or NIX, that bind to LC3/GABARAP proteins through an LIR motif [20], [21], [30], [31], [32], [37]. Consequently, our data indicate that region 1 of DOR protein contains a NES and an LIR motif, which participate in nucleocytoplasmic shuttling as well as in binding to LC3/GABARAP proteins and the induction of autophagosome formation. Whether the DOR mutations disrupt protein-protein interaction through conformational changes in the molecule, in addition, to the cancellation of LIR or NES signals, remains to be determined.

In addition to the autophagic defects, the L36A/L40A DOR mutant also showed defective transcriptional activity, which was displayed when DOR was fused to a Gal4 DNA-binding domain or when co-transfected with nuclear hormone receptors. In all, region 1 participates in, at least, three distinct functions: a) it contains a NES motif that allows nucleocytoplasmic shuttling; b) it contains a LIR motif that permits interaction with autophagic proteins LC3/GABARAP, and c) it permits transcriptional activation.

Region 2 comprises residues 66 to 112 in the human DOR protein, and the segment 87 to 111 shows an alpha-helix structure when analyzed by NMR. Mutation of the negatively charged amino acid residues 97 (glutamate) and 98 (aspartate) into lysine residues, caused a number of alterations in DOR function. The E97K/D98K DOR mutant showed the formation of aberrant autophagosomes under basal and autophagy activation conditions. Surprisingly, although region 2 does not contains a NES site, the E97K/D98K DOR mutant was partially prevented from exiting the nucleus in response to the activation of autophagy. Furthermore, the E97K/D98K DOR mutant showed a marked reduction in transcriptional activity, which was displayed when DOR was tethered to a Gal4 DNA-binding domain or when co-transfected with nuclear hormone receptors. Since D98 caps the N-terminal of the helix present in this region, its mutation may have induced a partial unfolding of the helix, reducing its ability to interact with transcriptional activators. The similarity between some of the alterations displayed by DOR mutants in regions 1 and 2, and specifically the alteration in autophagy-mediated nuclear exit by the region 2, in the absence of a NES motif, suggest intra-molecular cross talk between regions 1 and 2. This cross-talk may permit cooperation in the binding of partners relevant in autophagy or in transcriptional activation.

**Figure 12:**
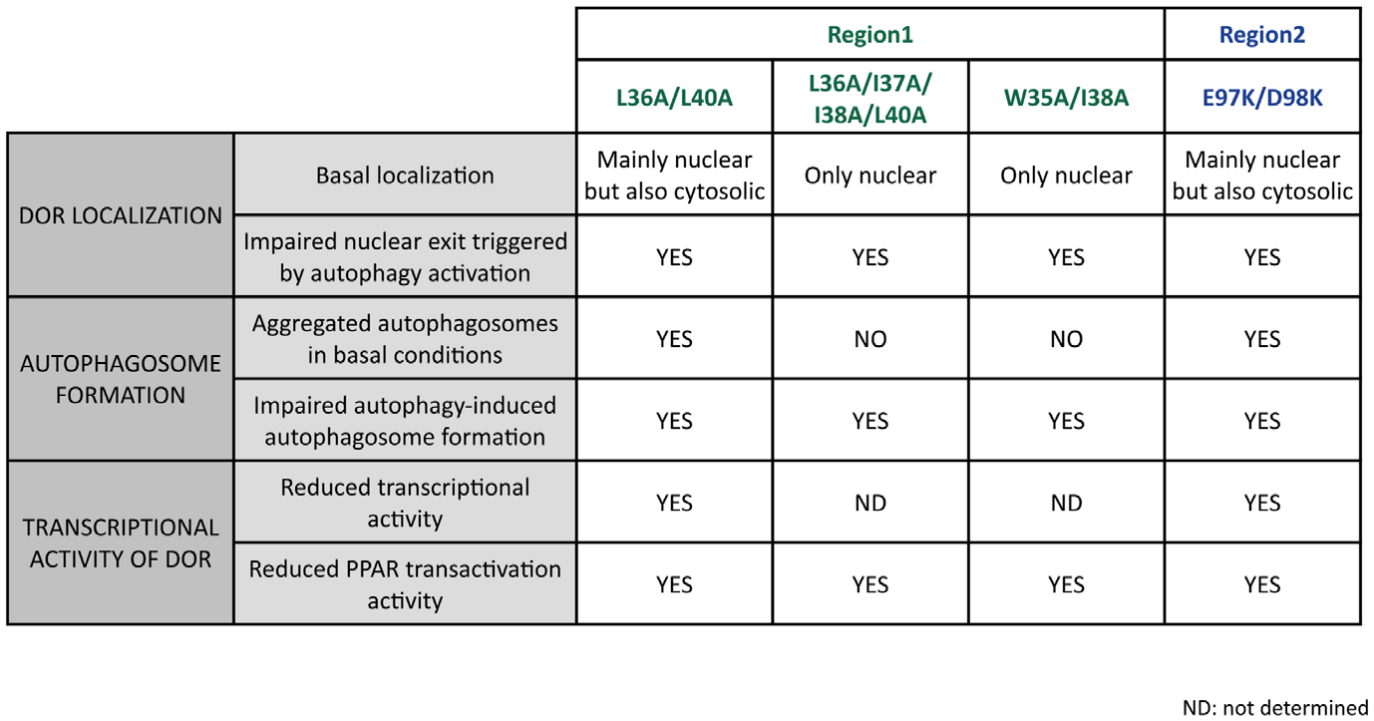
Overview of the effects of the studied mutations in DOR regions 1 and 2. Effects of the mutations on autophagosome formation, localization and transcriptional activity of the protein.

The overlap between the NES and LIR motifs in region 1 makes difficult to clearly differentiate the functional implications of each motif. Moreover, mutations in region 2 show similar alterations to DOR mutants in region 1, and more specifically of mutant L36/L40A. The comparison of the functional alterations detected on DOR localization, on autophagosome formation or on transcriptional activity is summarized in Figure 12. DOR mutants L36A/L40A and E97K/D98K show a basal nuclear localization similar to wild-type DOR and are located mainly in the nucleus but also in the cytosol. We believe that the copies of the mutant proteins that remain in the cytosol are the ones responsible for the formation of GFP-LC3 aggregates in basal conditions. This autophagosomal aggregation reflects a collapse of the autophagic machinery, which may also imply a sequestration of autophagic proteins. Under those conditions, mTOR-dependent pathways do not seem to be able to trigger an appropriate autophagy by dysregulated formation of autophagosomes and/or autophagolysosomes. However, NES (L36A/I37A/I38A/L40A) and LIR (W35A/I38A) DOR mutants stayed in the nucleus even in basal conditions and they did not show a collapse of the basal autophagic machinery but a reduced formation of autophagosomes (Figure 12). It is likely that the DOR mutant L36A/I37A/I38A/L40A shows cancellation of the NES motif as well as alterations as a LIR motif. We propose that the function of the LIR motif in autophagy is tightly linked to the localization of the protein. Moreover, our data suggest that the different activities of the protein DOR are subjected to intramolecular control. This requires a more detailed functional analysis of different mutations in the key motifs of DOR.

In all, our results reveal DOR and TP53INP1 as dual regulators of autophagy and transcription. Regarding transcriptional activity, both proteins can activate the transcription of a reporter gene for Gal4 when fused to a Gal4 DNA binding domain, but DOR coactivates nuclear hormone receptors while TP53INP1 transactivates p53 and p73. In addition, both proteins regulate positively autophagy. It will be relevant to further study the role of TP53INP1 on autophagy to elucidate whether they use different mechanisms compared to DOR.

## Supporting Information

Figure S1 **ClustalW multiple sequence alignment of DOR protein family members.** The two conserved regions, corresponding to amino acid residues 28–42 (region 1) and 66–112 (region 2) in human DOR protein, are marked with a red line (region 1) and a blue line (region 2) below the sequences. Exons are displayed in alternate black and blue. Amino acids in red indicate that their codons are split between exons. (*) Identity; (:) strongly similar; (.) weakly similar.
(PDF)

Table S1 **Sequences of DOR and TP53INP1 proteins used in this study.** List of DOR and TP53INP1 protein sequences with their common and scientific names. Exons are represented as alternating black and blue text. Amino acids shared by two exons (intron phases 1 or 2) are shown in red. Internal splicing amino acid in DOR in exon two is in phase 1 and is shown in brown. The exon structure of the sponge, rainbow-trout and salmon sequences are unresolved.
(PDF)

Table S2 **Table of sequence identities between the members of the gene family.** Identities between the DOR proteins (in green), TP53INP1 (in orange) and between ancestor DOR proteins (in yellow) 1, *Trichoplax adhaerens* DOR. 2, *Aplysia californica* DOR. 3, *Nematostella vectensis* DOR. 4, *Branchiostoma floridae* DOR. 5, *Strongylocentrotus purpuratus* DOR. 6, *Ciona intestinalis* DOR. 7, Capitella sp. DOR. 8, *Drosophila melanogaster* DOR. 9, *Oryzias latipes* TP53INP1. 10, *Callorhinchus milii* TP53INP1. 11, *Xenopus tropicalis* TP53INP1. 12, *Gallus gallus* TP53INP1. 13, *Anolis carolinensis* TP53INP1. 14, *Monodelphis domestica* TP53INP1. 15, *Mus musculus* TP53INP1. 16, *Homo sapiens* TP53INP1. 17, *Oryzias latipes* DOR. 18, *Callorhinchus milii* DOR. 19, *Xenopus tropicalis* DOR. 20, *Gallus gallus* DOR. 21, *Anolis carolinensis* DOR. 22, *Monodelphis domestica* DOR. 23, *Mus musculus* DOR. 24, *Homo sapiens* DOR.
(PDF)
