# Correction: *DOR/Tp53inp2* and *Tp53inp1* Constitute a Metazoan Gene Family Encoding Dual Regulators of Autophagy and Transcription

**DOI:** 10.1371/annotation/f2e121d7-0878-44b1-a5c2-994231626fe1

**Published:** 2012-05-07

**Authors:** Ana Sancho, Jordi Duran, Antonio García-España, Caroline Mauvezin, Endalkachew A. Alemu, Trond Lamark, Maria J. Macias, Rob DeSalle, Miriam Royo, David Sala, Javier U. Chicote, Manuel Palacín, Terje Johansen, Antonio Zorzano

Equal contributions designations were inadvertently omitted. The first three authors, Ana Sancho, Jordi Duran, and Antonio García-España, contributed equally to this work. 

