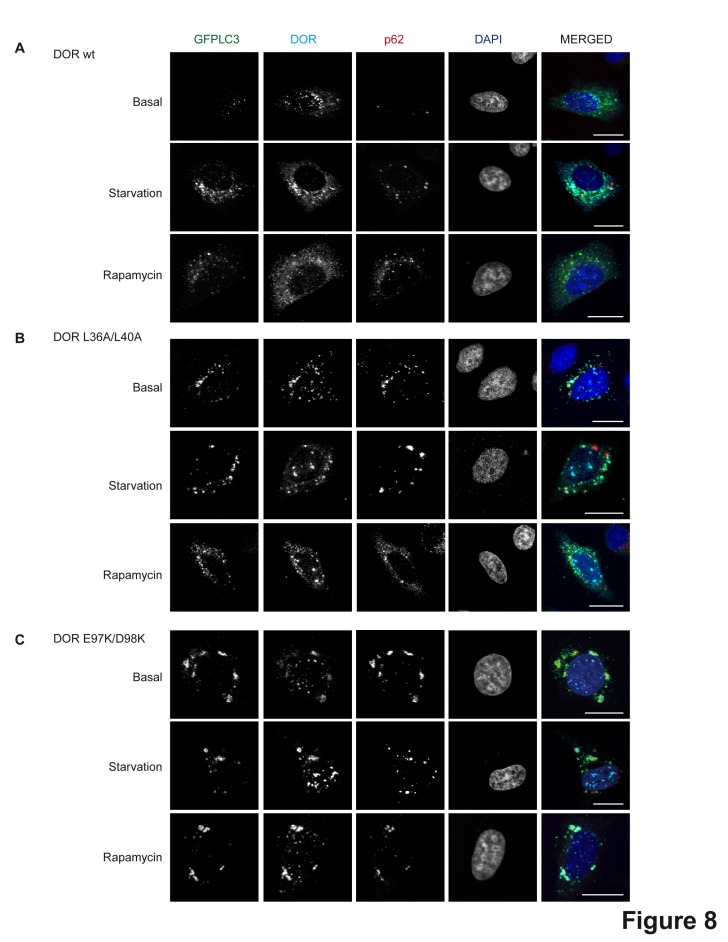# Correction: *DOR/Tp53inp2* and *Tp53inp1* Constitute a Metazoan Gene Family Encoding Dual Regulators of Autophagy and Transcription

**DOI:** 10.1371/annotation/4d84cc19-b887-4c1e-a26f-1968819f8c70

**Published:** 2012-06-29

**Authors:** Ana Sancho, Jordi Duran, Antonio García-España, Caroline Mauvezin, Endalkachew A. Alemu, Trond Lamark, Maria J. Macias, Rob DeSalle, Miriam Royo, David Sala, Javier U. Chicote, Manuel Palacín, Terje Johansen, Antonio Zorzano

Figure 8 is incomplete. Panels A, B, and C can be viewed here: 

**Figure:**