# Supplementary material for: DOR/Tp53inp2 and Tp53inp1 Constitute a Metazoan Gene Family Encoding Dual Regulators of Autophagy and Transcription
Source: PLoS One. 2012 Mar 28;7(3):e34034. doi: 10.1371/journal.pone.0034034 (PMC3314686; doi:10.1371/journal.pone.0034034)
Supplement: Figure S1 — ClustalW multiple sequence alignment of DOR protein family members. The two conserved regions, corresponding to amino acid residues 28–42 (region 1) and 66–112 (region 2) in human DOR protein, are marked with a red line (region 1) and a blue line (region 2) below the sequences. Exons are displayed in alternate black and blue. Amino acids in red indicate that their codons are split between exons. (*) Identity; (:) strongly similar; (.) weakly similar. (PDF) [file pone.0034034.s001.pdf]

```
DOR Human               -MFQRLSSLFFSTP-----------------SPPE-DPDCPRAFVS--E-----------------------------------------
DOR Opossum             -MFQRLTSLFFSTS-----------------PPSE-EPPSPKAFICPED-----------------------------------------
DOR Lizard              -MFQRLTSLFFSDS-----------------SAPE-DLEEPKPFIS--K-----------------------------------------
DOR Chicken             -MFQRLTSLFFSDS-----------------SAPE-GLEEPKPFVS--E-----------------------------------------
DOR Frog                -MFQRLTRLFFSDV-----------------PSS--NTNEPKPIIS--------------------------------------------
DOR Medaca              -MFQRLSNLLFGEV-----------------EEVAAELKGPNPCLT--------------------------------------------
DOR Elephant fish       -MFHRFTSLFYGGS-----------------ENT--CIEGPDPSLT--------------------------------------------
TP53INP1 Human          -MFQRLNKMFV---------------------GEVSSSS--NQEPEF-N-----------------------------------------
TP53INP1 Opossum        -MFQRLNSMFV---------------------GEIKNSS--SKEPEF-S-----------------------------------------
TP53INP1 Lizard         -MFQRLNNMFM---------------------EEINNLP--SQEPAF-S-----------------------------------------
TP53INP1 Chicken        -MFQRLNNMFM---------------------GEIDGLS--SQEPEF-S-----------------------------------------
TP53INP1 Frog           -MFQMLNNMFA---------------------RECSNAL--SQETKL-S-----------------------------------------
TP53INP1 Medaca         -MFQVFASALFRDG-----------------VEELSQCSRPGDDKRE-E-----------------------------------------
TP53INP1 Elephant fish  -MYQRFSSMLF---------------------GEIDGAERESQELEI-S-----------------------------------------
DOR Sea squirt          -MLNTIAYWAGFGS------------------NDTPVVDDNLSFIT--------------------------------------------
DOR Lancelet            -MFSAVTNLLFGASEEVG-----------------ACDLRTS------------------------------------------------
DOR Sea urchin          -MLSGLSDYLFGSSNNQAGD---------AMVTDDTQPQRDTPVKDPVK-----------------------------------------
DOR Fly                 -MLSSLASYLFGSATSDSISQEANPAQNRTNASNANSSSSPGPTSDPAAGDVIEVTSSTPSVAGSSRGAVRASNGKRGKNRRGKQQRTNQ
DOR Sea slug            -MFNSVAKYLWGDANEVEQG----------YMEVSGQADTAGEQLDL-AI----------------------------------------
DOR Capitella           -MLSGISSYLFG-------------------ASSAAEDNLPQPTVDPPE-----------------------------------------
DOR Sea anemone         -MFSSLTSYIWGQT------------------DDQVVPDCPLDVED--------------------------------------------
DOR Trychoplax          MLWQSLSSYLWDQ-------------------SSTEDASNHRVNVS--------------------------------------------
                         :   .

DOR Human               ----------------------EDEVDGWLIIDLPD------------------------------------------------------
DOR Opossum             ----------------------EEEVDGWLIIDLPD------------------------------------------------------
DOR Lizard              ----------------------EEEEDDWLIIDITGEKDS--------------VCAS--------------------------------
DOR Chicken             ----------------------EEEEDGWLIIELGA------------------------------------------------------
DOR Frog                ----------------------EEEDDGWLIIDIPESYDL--------------NSSGDE------------------------------
DOR Medaca              ----------------------EADEEGWMLVNLPD------------------------------------------------------
DOR Elephant fish       ----------------------EKEDDGWLIVDFPVEVKAS-------------TCVPSP------------------------------
TP53INP1 Human          ----------------------EKEDDEWILVDFIDTCTG-----------FSAEEEEEEE-----------------------------
TP53INP1 Opossum        ----------------------EKEDDEWILVDFIDTN-------------FSTEVEEE-------------------------------
TP53INP1 Lizard         ----------------------EKEEEEWIVVDFIDTCNN-----------FSMVEEEDEEEEEEEEDNC--------------------
TP53INP1 Chicken        ----------------------EKEDDEWILVDFIADT-C-----------TNCTTEQD-------------------------------
TP53INP1 Frog           ----------------------EKEDDEWILVDFIAQVDP-----------GSRVSEEAA------------------------------
TP53INP1 Medaca         ----------------------EEDDEDWILVNYLTDACSGDCGDGLSRSMLSPEDEDEEED----------------------------
TP53INP1 Elephant Fish  ----------------------EKEDEEWILVDYIDGM----------------------------------------------------
DOR Sea squirt          -----------------------KETGDWTLVDLENQND---------------------------------------------------
DOR Lancelet            -----------------------TLDDDWVLVELPAGMNIQQMEKE--------TEALT-------------------------------
DOR Sea urchin          ---------------------EIEMDGDWVLVDVTND-----------------SGSPS-------------------------------
DOR Fly                 QQRKQQPAITKLLTPSGEIVDEDFDEDEWYIVEKEDEEDD--------------SLPRSDSEEELSVVEVSQPRGGSNNASSPMVTVATG
DOR Sea slug            ----------------------HQDDDDWLVVGQPGASDIELDEAASLRCSYNPLTGCSDSVQGALQDCTMNLNN---------------
DOR Capitella           ----VQDDLERLMTR--------EGEEEWVLVDKAAR-----------------SPPRS-------------PR----------------
DOR Sea Anemone         ----------------------SRTEEGWIMVDLGSVVNPKD------------------------------------------------
DOR Trychoplax          ----------------------LTEEDDWILVVKKENKD---------------------------------------------------
                                                    * ::

DOR Human               --------SYAAP-------------------------------------PSPGAAPAP----AG-------------------RPPPAP
DOR Opossum             --------SFAAP-------------------------------------PSP----------K----------------------PASP
DOR Lizard              -----CCHHGSLP-------------------------------------PTSSLPLSPSDCISRSM-----------------PSQPDP
DOR Chicken             -------HTGS----------------------------------------------------S--T-----------------PTLPSP
DOR Frog                -----VAQEQEDD-------------------------------------ATPSPLPHSLADRIGWS-----------------IPPHPP
DOR Medaca              --------PGSEC-------------------------------------GGN----------GGRS-----------------REGSER
DOR Elephant fish       -----HLPDDHLP-------------------------------------LASSSLESLCHCGISSR-----------------PPRSKA
TP53INP1 Human          --DISEESPTEHP-------------------------------------SVFSCLPASLECLADTS--------------DSCFLQFES
TP53INP1 Opossum        --DVSEESPTNHP-------------------------------------PVFSCLPASLECLADTS--------------ESCFVQFEA
TP53INP1 Lizard         --NIHETSSVDHP-------------------------------------PVFSHLPNSLECLSDAS--------------ESCFIQFDS
TP53INP1 Chicken        --DIAETSPAGSS-------------------------------------PVFTCLPSPLEHLPEAG--------------ESCFIQFES
TP53INP1 Frog           --TFEVISHSDET-------------------------------------SVLPHVSNTFERLGTTS--------------DSCFIHFNL
TP53INP1 Medaca         --LVMIPSPIASP-------------------------------------AIRYPSCTSLNSVADTDPDGGVDEDEYVDDEEGGFLRLDA
TP53INP1 Elephant fish  -----DRATSPNS-------------------------------------AMFSSSTSSLELLGNSS--------------DPCFLQLDS
DOR Sea squirt          ----------AQS-------------------------------------NYPTPPPSP-------------------------------
DOR Lancelet            -----QSRPGDR--------------------------------------PSTS---SSPVPIPEN------------------------
DOR Sea urchin          -----PLRSPTRS-------------------------------------PSKDGFQSQPQAVPEKP-----------------------
DOR Fly                 TAFNCRRRQGVNSCSLYSGPRPQQQRNYLQRSRVSRPLSISTLSPPRSVPALGAGDHDTLTQSLYVASPSGSD-------QGQDHGQGAN
DOR Sea slug            FDLDSNYSSDGES---------------------------------GGDSGVRPSSPESVFSVCSGR---------------SHATYRPS
DOR Capitella           ----CRFR------G------------------------------SADVTPNGSSGHSTPVHILHPS-----------------------
DOR Sea anemone         ---------------------------------------------------------VKKHEKAEN---------------------AVP
DOR Trychoplax          ----------------------------------------------------LEEVSNEDQLVKNRK-----------------------
```

```
DOR Human                SLMDESWFVTPPACFTAEGPGLGPARLQSSPLEDLLIEHPSMSVYVTGS---------------------TIVLEPGS-PSPLPDA----
DOR Opossum              CLLDESWFVTPPACFTAEGPELGPAHLESSPLEDLLIEHPSMSVYVTGS---------------------TIVLEPGPGPTLTPPR----
DOR Lizard               CLMDESWFVTPPPCFTAECS--DPVSMESNPMEDLLIEHPSMSVYVTSS---------------------TIVVETQT---PEEH-----
DOR Chicken              CLMDESWFVTPPPCFTAEEP--GPDGVGSSPMEDLLIEHPSMSVYVTS----------------------TLELDAEG---PQD------
DOR Frog                 QSMDESWFVTPPPCFTAEAP--GQDELGTSPLEDLLIEHPSMSVYITNG---------------------SIVVEEDT---REAP-----
DOR Medaca               GCMDESWFVTPPPCFTAEGA-----TAEASPMEDLLIEHPSMSVYVSPNN--------------------TSMVSTSDLSVVGEEC----
DOR Elephant fish        CTLEESWFVTPPPCFTAEGQ--EPGEVEISPLENLLIEHPSMSVYAGSN---------------------TNISTM--------------
TP53INP1 Human           CPMEESWFITPPPCFTAGGL--TTIKVETSPMENLLIEHPSMSVYAVH----------------------NSCPGLSEATRGTD------
TP53INP1 Opossum         CPMEESWFITPPPCFTAGGL--TTIKVETSPLENLLIEHPSMSVY-------------------------NSSRNLNEAGCETD------
TP53INP1 Lizard          CPMEESWFITPPPCFTAGGL--ATLKVETSPLENLLIEHPSMSVYAVH----------------------NTHHSLNKTSCGDEEEEEED
TP53INP1 Chicken         CPMEESWFITPPPCFTAGGL--TAIKVETSPMENLLIEHPSMSVYAVH----------------------NACHSLSDTGCGDE------
TP53INP1 Frog            CPMEESWFVTPPPCFTAGEL--TSMAVKTSPMENLLIEHPSMSVYAVH----------------------NMCHKP-ETSCESG------
TP53INP1 Medaca          CSLEESWFVTPPPCFTGRGS--QPVLLETSPLENLLIEHPSMSVYAHHSPPRLMLNPLPQHSLEGKLDFLSSGPSDRKPSRGKEK-----
TP53INP1 Elephant fish   CALEESWFITPPPCFTAGGQ--APVQVEMSPMENLLIEHPSMSVYTVH------------------------------------------
DOR Sea squirt           -RIEHSWLITPPPCFTAEAS---MHVGALNARENLLIEHPSMFVKPAE-----------------------------------P------
DOR Lancelet             HPVEESWFVTPPPCFTAGGQ--SPVAMETNPMENLLIEHPSMSVYVQ---------------------QRSS----RNSSGETTE----
DOR Sea urchin           HRMEESWFITPPPCFTAGGH--SPHQLATSPLEDLLIEHPSMSVYHHP-------------------SQRGAPMAHRRQHLPRQK-----
DOR Fly                  VLMEESWYVTPPPCFTSIGP----INMETSPFENLLIEHPSMSVYHSIRST--------Q---EGTDSFVNLDLGVSTEVPQQRE-----
DOR Sea slug             GSSHDPWIVAPPPCFTGSGLG-ELPTISSSPLENLLIEHPSMSVYLSVPPS--------SLPPSHPFHLAHLAGESAPASSEPRDSGDDS
DOR Capitella            -LCES-WLVTPPSCFTAAG------SARSDPLENLLIEHPSMSVYS----------------------LG-QRGGRGRSAGEESE-----
DOR Sea anemone          DSVEESWYVTPPSCFESRDN--FSGVLESNPMEDLLIEHPSMSVYGP-----------------------RRSADASAGPSSRS------
DOR Trychoplax           -EKESGWLLTPPPCFDGHVM----EELSVSPFEDLLIEHPSMSIYRR--------------------------IDSSASIRNN-------
                            .  * ::**.** .            .. *:******** :
```

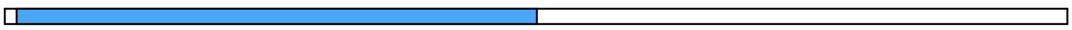

```
DOR Human                ----ALPDGDLSEGELTPARREPRAARHAAPLPAR-----AALLEKAGQVR---RLQRARQRAERHALSAKAVQRQNRARESRPRRSKN-
DOR Opossum              ----SPPPARAGHREVMQPRRDPGPRHHTASMPSR-----TALLEKASQAR---WVQRARQRADRQRLSPKVVQRQNRVRERHPRRSKH-
DOR Lizard               -----I----NRDGEEPERRLQRHAPHHSTSLATK-----AAILEKVSQVH---RIQRAKQLAEKHNFSQKVMQRQNRTRECRPRRAKH-
DOR Chicken              ----------DAVGEVPEPRLERHVPHRSASLSVK-----AAVLEKVSQAR---RVQRAKQLAEKPWLSQKALQRQDRARQRPPRRARQR
DOR Frog                 -----------RDRSPSKTRVERRTPHHATSMSAK-----AAILGKVGQAS---RIQRAKAHVDRRKISRKSLQRQNLAREIQGRSMTR-
DOR Medaca               ----ILSLASSVSSRVSEPGVIPAARSTMPTRVTRGGAAHPGALAKVTQVA---RVQRCKARIDRRHLSRNHIQRQNRTREQVPCHAAHA
DOR Elephant fish        -------------TESPARVSHHIRCYAAALAAH-----TSFLEQANQIR---HSQRAKEHLEKHLLNRNNIRRQNLIRESSSRQTKH-
TP53INP1 Human           ----ELHSPSSPRVEAQN-EMGQHIHCYVAALAAH-----TTFLEQPKSFR---PSQWIKEHSERQPLNRNSLRRQNLTRDCHPRQVKH-
TP53INP1 Opossum         ----EFHNPSSPRLEVQN-EMGQHVHCYVAALAAH-----STFLEQTKSFR---PTQWIKEHSERHSLNRNSLRRQNLTRDCHSRQIKH-
TP53INP1 Lizard          EEEKENRNATNPQSEAQS-QMGQHIRCYIATLATH-----STFLEQTKNLR---PSQWTKEHHERQYLTRNCLRRQNLTRDCYSRQLKN-
TP53INP1 Chicken         ----EFCGPGSPRLEARN-ETGQRVHCFVAALPTR-----SSVLEKNKSFR---PTHWIKEHGERHYPSRNGLRRQNLTRDCHSRHIKH-
TP53INP1 Frog            -----FPSPDRTELATENKKKGKHIHCSIAALAAR-----MKGLENTKIYL---GDKLTKLHLEKHP-SRKGFRRQNLIRGCRSQQTKH-
TP53INP1 Medaca          VRCSIEGSRHRQDVAAAQRRPNLHSPCYAATLSPN-----AGFLQQQQRRP---GS----AAQRSQPLSRKGLRRRNLLR-----PPKT-
TP53INP1 Elephant fish   --NIHSSLRENSRVIPIVQYVPRRVSAFRIRDSVL-----EHTNHIHFMQC-------AKLYVERRKLSCNHLRRQNRARKRYFAKEKH-
DOR Sea squirt           ----------SEEPKNKTESRSVQRHKGAPTSLR--------RSHRRASR---QASCVISQMHRSNQIKRDVARSNKAMQK--------
DOR Lancelet             ------SSDSSGQHQQQRRDVVHRPPQRAAAIAAR----VGIVEQA-KMKR---HAQRVQERHEKKANSKTNLQRANLTSCRPIRASR-H
DOR Sea urchin           ------SSRTVPKRTHNLRVLNAKPPRRAAAIAAR----VGIEQSANQVGL---QEQRDTKRHQQRQYSPGKIERHNKAYHHQSASSRRH
DOR Fly                  ------EPEPEAEPQDQRQALQEQQRAPNARFDSH----AAVQLKQQTLAR---QSQKSKNKKEHQQLCRSAIKRANKVRDFQAKANRPR
DOR Sea slug             GEPGDALREDRTHQLPVGEQLPAAHRAHREGLGRHWGFAAPAQVSPAQAVARLHAAQRVQSGKSNKAVSRQKCRRENKVYEYKGCPKGNT
DOR Capitella            ------ESDVEEEAVRS-QRLQASRPHPLAINTAI----ASRKVTQR-------SMQKAVKKHEQQRLARNVMQRNNRVQKHNNSCRRPR
DOR Sea anemone          ----------ASQAAEENQELEKKPPRRTVQFQEK-----LELIAQKRKVE---APQHGTIRPNKKNIKKQNLVQFQHSRTRRTKKRDRM
DOR Trychoplax           ---------LDHDFAASLANSGDNNTHEDTHEQSR-----SRSIELYMDR----ELNKKSDRFNYIRKARDLNVKRSLTFSKAN--SRSY
                                                                                   : .
```

```
DOR Human                QSSFIYQPCQRQFNY-------
DOR Opossum              QGSFLHQPCQRQFNY-------
DOR Lizard               QGSFVYQPSQRQYNY-------
DOR Chicken              AGSFLHQPCQRHCNY-------
DOR Frog                 HRSFLCQPRQRQCNY-------
DOR Medaca               RNTFLHQPSKRNFCH-------
DOR Elephant fish        SGHVLHQPCQRQYN-------
TP53INP1 Human           NGWVVHQPCPRQYNY-------
TP53INP1 Opossum         NGWVVHQPCQRQYNY-------
TP53INP1 Lizard          NGILVHQPCQRQYNY-------
TP53INP1 Chicken         NGLFVHQPCQRQFNY-------
TP53INP1 Frog            SRLLVHQPSPRQYNY-------
TP53INP1 Medaca          GTMHLQQPIQRHLNF-------
TP53INP1 Elephant fish   FGHFKQPCQRNHKY--------
DOR Sea squirt           MRQIVHQPRKRC----------
DOR Lancelet             NRNRGLMCKQPQGRVNKGRC--
DOR Sea urchin           YKQNSSISGRHSGAIYKQPR--
DOR Fly                  RSEMQHCKLVSGANNNRSKCCY
DOR Sea slug             KRSKRSRPSSCKSGRMCQRV--
DOR Capitella            RS--ERMMQPSGRSNNRSMLH-
DOR Sea anemone          VGKHIGVHGKRGS---------
DOR Trychoplax           SNSWSRRHCFTNFSYRR-----
```

Supplementary Figure 1
