# Supplementary material for: DOR/Tp53inp2 and Tp53inp1 Constitute a Metazoan Gene Family Encoding Dual Regulators of Autophagy and Transcription
Source: PLoS One. 2012 Mar 28;7(3):e34034. doi: 10.1371/journal.pone.0034034 (PMC3314686; doi:10.1371/journal.pone.0034034)
Supplement: Table S1 — Sequences of DOR and TP53INP1 proteins used in this study. List of DOR and TP53INP1 protein sequences with their common and scientific names. Exons are represented as alternating black and blue text. Amino acids shared by two exons (intron phases 1 or 2) are shown in red. Internal splicing amino acid in DOR in exon two is in phase 1 and is shown in brown. The exon structure of the sponge, rainbow-trout and salmon sequences are unresolved. (PDF) [file pone.0034034.s002.pdf]

DOR Human [Homo sapiens] NP_067025
MFQRLSSLFFSTPSPPEDPDCPRAFVSEEDEVDGWLIIDLPDSYAAPPSPGAAPAPAGRP
PPAPSLMDESWFVTPPACFTAEGPGLGPARLQSSPLEDLLIEHPSMSVYVTGSTIVLEPG
SPSPLPDAALPDGDLSEGELTPARREPRAARHAAPLPARAALLEKAGQVRRLQRARQRAE
RHALSAKAVQRQNRARESRPRRSKNQSSFIYQPCQRQFNY

TP53INP1 Human [Homo sapiens] NP_150601
MFQRLNKMFVGEVSSSSNQEPEFNEKEDDEWILVDFIDTCTGFSAEEEEEEEDISEESPT
EHPSVFSCLPASLECLADTSDSCFLQFESCPMEESWFITPPPCFTAGGLTTIKVETSPME
NLLIEHPSMSVYAVHNSCPGLSEATRGTDELHSPSSPRVEAQNEMGQHIHCYVAALAAHT
TFLEQPKSFRPSQWIKEHSERQPLNRNSLRRQNLTRDCHPRQVKHNGWVVHQPCPRQYNY

DOR Opossum [Monodelphis domestica] XP_001371459
MFQRLTSLFFSTSPPSEEPPSPKAFICPEDEEEVDGWLIIDLPDSFAAPPSPKPASPCLL
DESWFVTPPACFTAEGPELGPAHLESSPLEDLLIEHPSMSVYVTGSTIVLEPGPGPTLTP
PRSPPPARAGHREVMQPRRDPGPRHHTASMPSRTALLEKASQARWVQRARQRADRQRLSP
KVVQRQNRVRERHPRRSKHQGSFLHQPCQRQFNY

TP53INP1 Opossum [Monodelphis domestica] XP_001368955
MFQRLNSMFVGEIKNSSSKEPEFSEKEDDEWILVDFIDTNFSTEVEEEDVSEESPTNHPP
VFSCLPASLECLADTSESCFVQFEACPMEESWFITPPPCFTAGGLTTIKVETSPLENLLI
EHPSMSVYNSSRNLNEAGCETDEFHNPSSPRLEVQNEMGQHVHCYVAALAAHSTFLEQTK
SFRPTQWIKEHSERHSLNRNSLRRQNLTRDCHSRQIKHNGWVVHQPCQRQYNY

DOR Lizard [Anolis carolinensis] Genomic profiling
MFQRLTSLFFSDSSAPEDLEEPKPFISKEEEEDDWLIIDIT**G**EKDSVCASCCHHGSLPPT
SSLPLSPSDCISRSMPSQPDPCLMDESWFVTPPPCFTAECSDPVSMESNPMEDLLIEHPS
MSVYVTSSTIVVETQTPEEHINRD**G**EEPERRLQRHAPHHSTSLATKAAILEKVSQVHRIQ
RAKQLAEKHNFSQKVMQRQNRTRECRPRRAKHQGSFVYQPSQRQYNY

TP53INP1 Lizard [Anolis carolinensis] Genomic profiling
MFQRLNNMFMEEINNLPSQEPAFSEKEEEEWIVVDFI**D**TCNNFSMVEEEDEEEEEEEEDN
CNIHETSSVDHPPVFSHLPNSLECLSDASESCFIQFDSCPMEESWFITPPPCFTAGGLAT
LKVETSPLENLLIEHPSMSVYAVHNTHHSLNKTSCGDEEEEEEDEEEKENRNATNP**Q**SEA
QSQMGQHIRCYIATLATHSTFLEQTKNLRPSQWTKEHHERQYLTRNCLRRQNLTRDCYSR
QLKNNGILVHQPCQRQYNY

DOR Chicken [Gallus gallus] CN231263, XP_001232258 and
genomic profiling
MFQRLTSLFFSDSSAPEGLEEPKPFVSEEEEEDGWLIIELGAHTGSSTPTLPSPCLMDES
WFVTPPPCFTAEEPGPDGVGSSPMEDLLIEHPSMSVYVTSTLELDAEGPQDDAVGEVPEP
RLERHVPHRSASLSVKAAVLEKVSQARRVQRAKQLAEKPWLSQKALQRQDRARQRPPRRA
RQRAGSFLHQPCQRHCNY

TP53INP1 Chicken [Gallus gallus] NP_001026117
MFQRLNNMFMGEIDGLSSQEPEFSEKEDDEWILVDFIADTCTNCTTEQDDIAETSPAGSS
PVFTCLPSPLEHLPEAGESCFIQFESCPMEESWFITPPPCFTAGGLTAIKVETSPMENLL
IEHPSMSVYAVHNACHSLSDTGCGDEEFCGPGSPRLEARNETGQRVHCFVAALPTRSSVL
EKNKSFRPTHWIKEHGERHYPSRNGLRRQNLTRDCHSRHIKHNGLFVHQPCQRQFNY

DOR Frog [Xenopus tropicalis] multiple ESTs short and long
MFQRLTRLFFSDVPSSNTNEPKPIISEEEDDGWLIIDIPESYDLNSSGDEVAQEQEDDAT
PSPLPHSLADRIGWTP53INP1PHPPQSMDESWFVTPPPCFTAEAPGQDELGTSPLEDLL
IEHPSMSVYITNGSIVVEEDTREAPRDRSPSKTRVERRTPHHATSMSAKAAILGKVGQAS
RIQRAKAHVDRRKISRKSLQRQNLAREIQGRSMTRHRSFLCQPRQRQCNY

TP53INP1 Frog [Xenopus tropicalis] ensemble
ENSXETP00000014600 and ESTs
MFQMLNNMFARECSNALSQETKLSEKEDDEWILVDFIAQVDPGSRVSEEAATFEVISHSD
ETSVLPHVSNTFERLGTTSDSCFIHFNLCPMEESWFVTPPPCFTAGELTSMAVKTSPMEN
LLIEHPSMSVYAVHNMCHKPETSCESGFPSPDRTELATENKKKGKHIHCSIAALAARMKG
LENTKIYLGDKLTKLHLEKHPSRKGFRRQNLIRGCRSQQTKHSRLLVHQPSPRQYNY

DOR Medaca [Oryzias latipes] BJ721597 genomic profiling
MFQRLSNLLFGEVEEVAAELKGPNPCLTEADEEGWMLVNLPDPGSECGGNGGRSREGSER
GCMDESWFVTPPPCFTAEGATAEASPMEDLLIEHPSMSVYYVSPNNTSMVSTSDLSVVGEE
CILSLASSVSSRVSEPGVIPAARSTMPTRVTRGGAAHPGALAKVTQVARVQRCKARIDRR
HLSRNHIQRQNRTREQVPCHAAHARNTFLHQPSKRNFCH

TP53INP1 Medaca [Oryzias latipes]genomic profiling and ESTS
(AM335308;AM316165; AM322529; AM306335)
MFQVFASALFRDGVEELSQCSRPGDDKREEEEDDEDWILVNYLTDACSGDCGDGLSRSML
SPEDEDEEEDLVMIPSPIASPAIRYPSCTSLNSVADTDPDGGVDEDEYVDDEEGGFLRLD
ACSLEESWFVTPPPCFTGRGSQPVLLETSPLENLLIEHPSMSVYAHHSPPRLMLNPLPQH
SLEGKLDFLSSGPSDRKPSRGKEKVRCSIEGSRHRQDVAAAQRRPNLHSPCYAATLSPNA
GFLQQQQRRPGSAAQRSQPLSRKGLRRRNLLRPPKTGTMHLQQPIQRHLNF

DOR Elephant fish [Callorhinchus milii] genomic profiling
MFHRFTSLFYGGSENTCIEGPDPSLTEKEDDGWLIVDFPVE**V**KASTCVPSPHLPDDHLPL
ASSSLESLCHCGISSRPPRSKACTLEESWFVTPPPCFTAE**G**QEPGEVEISPLENLLIEHP
SMSVYAGSNTNISTMEDEPSEVPSS**R**SARQPRAERLTAPSPAVAAGIQSSLVEKVSQVRR
LQRAKQRMERHQLSRNRIQRQNLTRECRLHRTKHHGSFVYQPRQRHCNY

TP53INP1 Elephant fish [Callorhinchus milii] genomic
profiling
MYQRFSSMLFGEIDGAERESQELEISEKEDEEWILVDYI**D**GMDRATSPNSAMFSSSTSSL
ELLGNSSDPCFLQLDSCALEESWFITPPPCFTAGGQAPVQVEMSPMENLLIEHPSMSVYT
VHNIHSSLRENS**R**VIPIVQYVPRRVSAFRIRDSVLEHTNHIHFMQCAKLYVERRKLSCNH
LRRQNRARKRYFAKEKHFGHFKQPCQRNHKY

DOR Sea squirt [Ciona intestinalis] XP_002130814
MLNTIAYWAGFGSNDTPVVDDNLSFITKETGDWTLVDLENQN**D**AQSNYPTPPPSPRIEHS
WLITPPPCFTA**E**ASMHVGALNARENLLIEHPSMFVKPAEPSEEPKNKTESRSVQRHK**G**AP
TSLRRSHRRASRQASCVISQMHRSNQIKRDVARSNKAMQKMRQIVHQPRKRC

DOR Lancelet [Branchiostoma floridae] CF918976; BW697867;
CF9189763; BW698074;
MFSAVTNLLFGASEEVGACDLRTSTLDDDWVLVELPAGMNIQQMEKETEALTQSRPGDRP
STSSSPVPIPENHPVEESWFVTPPPCFTAGGQSPVAMETNPMENLLIEHPSMSVYVQQRS
SRNSSGETTESSDSSGQHQQQRRDVVHRPPQRAAAIAARVGIVEQAKMKRHAQRVQERHE
KKANSKTNLQRANLTSCRPIRASRHNRNRGLMCKQPQGRVNKGRC

DOR Sea urchin [Strongylocentrotus purpuratus] XP_001177555
NCBI predicted plus genomic profiling and multiple ESTs
MLSGLSDYLFGSSNNQAGDAMVTDDTQPQRDTPVKDPVKEIEMDGDWVLVDVTNDSGSPS
PLRSPTRSPSKDGFQSQPQAVPEKPHRMEESWFITPPPCFTAGGHSPHQLATSPLEDLLI
EHPSMSVYHHPSQRGAPMAHRRQHLPRQKSSRTVPKRTHNLRVLNAKPPRRAAAIAARVG
IEQSANQVGLQEQRDTKRHQQRQYSPGKIERHNKAYHHQSASSRRHYKQNSSISGRHSGA
IYKQPR

DOR Fly Drosophila melanogaster] NP_728991
MLSSLASYLFGSATSDSISQEANPAQNRTNASNANSSSSPGPTSDPAAGDVIEVTSSTPS
VAGSSRGAVRASNGKRGKNRRGKQQRTNQQQRKQQPAITKLLTPSGEIVDEDFDEDEWYI
VEKEDEEDDSLPRSDSEEELSVVEVSQPRGGSNNASSPMVTVATGTAFNCRRRQGVNSCS
LYSGPRPQQQRNYLQRSRVSRPLSISTLSPPRSVPALGAGDHDTLTQSLYVASPSGSDQG
QDHGQGANVLMEESWYVTPPPCFTSIGPINMETSPFENLLIEHPSMSVYHSIRSTQEGTD
SFVNLDLGVSTEVPQQREEPEPEAEPQDQRQALQEQQRAPNARFDSHAAVQLKQQTLARQ
SQKSKNKKEHQQLCRSAIKRANKVRDFQAKANRPRRSEMQHCKLVSGANNNRSKCCY

DOR Sea slug [Aplysia californica] genomic profiling
MFNSVAKYLWGDANEVEQGYMEVSGQADTAGEQLDLAIHQDDDDWLVVGQPGASDIELDE
AASLRCSYNPLTGCSDSVQGALQDCTMNLNNFDLDSNYSSDGESGGDSGVRPSSPESVFS
VCSGRSHATYRPSGSSHDPWIVAPPPCFTGSGLGELPTISSSPLENLLIEHPSMSVYLSV
PPSSLPPSHPFHLAHLAGESAPASSEPRDSGDDSGEPGDALREDRTHQLPVGEQLPAAHR
AHREGLGRHWGFAAPAQVSPAQAVARLHAAQRVQSGKSNKAVSRQKCRRENKVYEYKGCP
KGNTKRSKRSRPSSCKSGRMCQRV

DOR Capitella [Capitella sp] EY564360 and genomic profiling
MLSGISSYLFGASSAAEDNLPQPTVDPPEVQDDLERLMTREGEEEWVLVDKAA**R**SPPRSP
RCRFRGSADVTPNGSSGHSTPVHILHPSLCESWLVTPPSCFTAAGSARSDPLENLLIEHP
SMSVYSLGQRGGRGRSAGEESEESDVEEEAVRSQRLQASRPHPLAINTAIASRKVTQRSM
QKAVKKHEQQRLARNVMQRNNRVQKHNNSCRRPRRSERMMQPSGRSNNRSMLH

DOR Sea anemone [Nematostella vectensis] XP_001623518
MFSSLTSYIWGQTDDQVVPDCPLDVEDSRTEEGWIMVDLGSVVNPKDVKKHEKAENAVPD
SVEESWYVTPPSCFESRDNFSGVLESNPMEDLLIEHPSMSVYGPRRSADASAGPSSRSAS
QAAEENQELEKKPPRRTVQFQEKLELIAQKRKVEAPQHGTIRPNKKNIKKQNLVQFQHSR
TRRTKKRDRMVGKHIGVHGKRGS

DOR Trichoplax [Trichoplax adherens] genomic profiling
MLWQSLSSYLWDQSSTEDASNHRVNVSLTEEDDWILVVKKENK**D**LEEVSNEDQLVKNRKE
KESGWLLTPPPCFDGHVMEELSVSPFEDLLIEHPSMSIYRRIDSSASIRNNLDHDFAASL
ANSGDNNTHEDTHEQSRSRSIELYMDRELNKKSDRFNYIRKARDLNVKRSLTFSKANSRS
YSNSWSRRHCFTNFSYRR

DOR Sponge [Ephydatia muelleri] AM760558
NLIWGYPAAESREEPTDHITVEAEDWLLVQRSDKAQVCEGGSAPLDHGSQVTSDNESVCS
DASWVVAPAPRFRDSPETTNAGHPLENLLIEHPAMSVYGQIQVGSDTIVPTADVQLVTTV
SEQAGQAPNRQRRQVALHMDVPPKMPIAATQRVQFKHPKLSRASITRHNATVTKTRVPRM
RTRMSNSKKAGRRGC

DOR Rainbow trout [Oncorhynchus mykiss] CA358690
MFQALSSLFFGEVEEVAAEIKGPNPCVTDADEEGWMLVNLPEGATAETSPMEDLLIEHP
SMSVYVSPSNLSMISNGNLSIVGEESITSLTGSMSRVAEPAATSAVHNTMPTRVTRGAA
AQAGTLAKVTQVARVQRGKARTERRHLGRNRIQRQNRTREQVPRHAAHARNTYLHQPCQ
RNICH

DOR Salmon [Salmo salar] BG935547
MIGKIFAHFLGSTDDDFPTDDNETYEELIEFEEGGWVVINIQENNSLTPPEEDPLENLL
IEHPSMSVYQIRHQTSDEVDADEEDASPRPVPVKQHVSWRLAAWGSPLPCNVQLLAIQR
ARTHMECKELTRSALQRQNLAKVRFSPSDRRFGHFKQPCQRLYNY

Supplementary table 1
