# Supplementary material for: DOR/Tp53inp2 and Tp53inp1 Constitute a Metazoan Gene Family Encoding Dual Regulators of Autophagy and Transcription
Source: PLoS One. 2012 Mar 28;7(3):e34034. doi: 10.1371/journal.pone.0034034 (PMC3314686; doi:10.1371/journal.pone.0034034)
Supplement: Table S2 — Table of sequence identities between the members of the gene family. Identities between the DOR proteins (in green), TP53INP1 (in orange) and between ancestor DOR proteins (in yellow) 1, Trichoplax adhaerens DOR. 2, Aplysia californica DOR. 3, Nematostella vectensis DOR. 4, Branchiostoma floridae DOR. 5, Strongylocentrotus purpuratus DOR. 6, Ciona intestinalis DOR. 7, Capitella sp. DOR. 8, Drosophila melanogaster DOR. 9, Oryzias latipes TP53INP1. 10, Callorhinchus milii TP53INP1. 11, Xenopus tropicalis TP53INP1. 12, Gallus gallus TP53INP1. 13, Anolis carolinensis TP53INP1. 14, Monodelphis domestica TP53INP1. 15, Mus musculus TP53INP1. 16, Homo sapiens TP53INP1. 17, Oryzias latipes DOR. 18, Callorhinchus milii DOR. 19, Xenopus tropicalis DOR. 20, Gallus gallus DOR. 21, Anolis carolinensis DOR. 22, Monodelphis domestica DOR. 23, Mus musculus DOR. 24, Homo sapiens DOR. (PDF) [file pone.0034034.s003.pdf]

1	2	3	4	5	6	7	8	9	10	11	12	13	14	15	16	17	18	19	20	21	22	23	24		
	24	20	20	22	16	20	23	18	22	17	20	21	24	19	20	16	18	20	23	19	11	20	14	**1**	DOR T. adhaerens
		20	31	20	20	24	20	17	22	23	20	17	22	21	18	26	25	25	22	21	24	25	27	**2**	DOR A. californica
			30	25	18	28	25	21	22	22	22	19	20	20	19	24	22	19	23	21	26	25	25	**3**	DOR N. vectensis
				36	24	28	28	24	33	28	33	28	30	29	31	29	32	28	31	32	24	28	28	**4**	DOR B. floridae
					27	30	27	24	27	19	24	20	21	22	20	24	27	28	29	24	26	23	26	**5**	DOR S. purpuratus
						26	23	23	23	26	25	27	23	28	28	22	29	25	25	25	25	20	20	**6**	DOR C. intestinalis
							33	20	27	21	18	23	23	24	24	26	25	23	32	28	26	24	26	**7**	DOR Capitella sp.
								23	28	24	24	25	21	25	27	25	26	22	33	27	24	26	28	**8**	DOR D. melanogaster
									36	29	28	28	33	32	32	24	31	24	27	27	32	28	27	**9**	TP53INP1 O. latipes
										45	52	55	53	56	56	34	41	31	35	36	36	34	35	**10**	TP53INP1 C. milii
											50	51	48	51	50	26	34	33	30	34	22	30	27	**11**	TP53INP1 X. tropicalis
												64	70	66	66	36	40	32	38	34	37	35	36	**12**	TP53INP1 G. gallus
													69	68	67	30	38	32	33	36	34	31	30	**13**	TP53INP1 A. carolinensis
														78	83	31	39	32	38	35	34	32	31	**14**	TP53INP1 M. domestica
															87	31	38	33	38	33	34	31	30	**15**	TP53INP1 M. musculus
																33	39	33	37	34	34	32	32	**16**	TP53INP1 H. sapiens
																	36	36	38	34	39	36	38	**17**	DOR O. latipes
																		41	42	48	41	43	41	**18**	DOR C. milii
																			55	51	46	45	46	**19**	DOR X. tropicalis
																				65	57	54	56	**20**	DOR G. gallus
																					52	52	53	**21**	DOR A. carolinensis
																						62	67	**22**	DOR M. domestica
																							85	**23**	DOR M. musculus
																								**24**	DOR H. sapiens

Supplementary table 2
